# Supplementary material for: Adolescents and age of consent to HIV testing: an updated review of national policies in sub-Saharan Africa
Source: BMJ Open. 2021 Sep 6;11(9):e049673. doi: 10.1136/bmjopen-2021-049673 (PMC8442095; doi:10.1136/bmjopen-2021-049673)
Supplement: Supplementary data [file bmjopen-2021-049673supp001.pdf]

Search Strategy

The process for collecting HTS guidelines included the following methods:

1. Search of internal WHO database for HIV Testing Services (HTS) policy documents

2. Country by country search of AIDSFree HTS policy database (https://aidsfree.usaid.gov/resources/guidance-data/hts)

3. Country by country search of IAPAC/HIV Policy Watch website (http://www.hivpolicywatch.org/database.html)

4. Broad Google search for HTS policies from each WHO country using the following key words
in English, French, Portuguese, and Spanish:
- country name AND “HIV testing” AND policy
- country name AND “HIV testing” AND guideline
- country name AND PrEP AND policy
- country name AND PrEP AND guideline
- country name AND “pre-exposure prophylaxis” AND policy
- country name AND “pre-exposure prophylaxis” AND guideline

The policy repository is maintained by WHO.
