# Supplementary material for: Adolescents and age of consent to HIV testing: an updated review of national policies in sub-Saharan Africa
Source: BMJ Open. 2021 Sep 6;11(9):e049673. doi: 10.1136/bmjopen-2021-049673 (PMC8442095; doi:10.1136/bmjopen-2021-049673)
Supplement: Supplementary data [file bmjopen-2021-049673supp002.pdf]

Policy data extraction tool

Two reviewers extracted policy data using a standardised tool. The tool included the following:

1. Country name
2. WHO priority country (yes/no)
3. Sub-region (ESA, WCA)
4. HIV testing policy identified? /yes/no, for HIVST policies, document separately)
   - Type of HIV testing policy document (HTS, NSP, ART, PMTCT, other)
   - Document name, primary data source
   - Notes about document and or secondary data sources
   - HIV prevalence (≥5%, <5%)
   - Year of publication
   - Language
5. HTS policy classification
   - Law or policy existing for HTS, age of consent clearly defined, including no age limit (0/1)
   - Law or policy existing for HTS, but no age of consent specified (0/1)
   - Law or policy existing for HTS, but no mention of 'adolescent' or 'minor' (0/1)
   - No law or policy for HTS
   - Age of minor not defined
   - No age limit – all or most adolescents eligible
6. Age of consent for HIV testing (including policy page reference)
7. Exceptions
   - Shows maturity and understanding of the process and potential results, health-care provider discretion
   - At risk of contracting HIV
   - Symptomatic
   - Pregnant
   - Parent, adolescent is already a parent
   - Head of household
   - Married
   - Commercial sex worker
   - Street child
   - Emancipated minor
   - Notes concerning exceptions

8. HIVST policy identified? (yes/no)
   - Type of HIVST policy doc (HTS, NSP, ART, other)
   - HIVST Document name (primary HIVST data source)
   - Notes about HIVST document and or secondary HIVST data source
   - Year of publication
   - Language
9. HIVST policy classification
   - HIVST law or policy existing, age of consent clearly defined (including no age limit)
   - HIVST law or policy existing, but no age of consent specified for HIVST
   - HIVST law or policy existing, but no mention of 'adolescent' or 'minor'
   - No law or policy for HIVST
   - No age limit – all or most adolescents eligible for HIVST
10. Age of consent for HIVST (including page reference)
11. Notes and exceptions for age of consent to HIVST
12. Age of consent as reported to NCPI (http://lawsandpolicies.unaids.org/)
