# Supplementary material for: Adolescents and age of consent to HIV testing: an updated review of national policies in sub-Saharan Africa
Source: BMJ Open. 2021 Sep 6;11(9):e049673. doi: 10.1136/bmjopen-2021-049673 (PMC8442095; doi:10.1136/bmjopen-2021-049673)

## SUPPLEMENTARY FILES

### Included policies

**Age of consent to HIV testing policies in 49 sub-Saharan African countries**

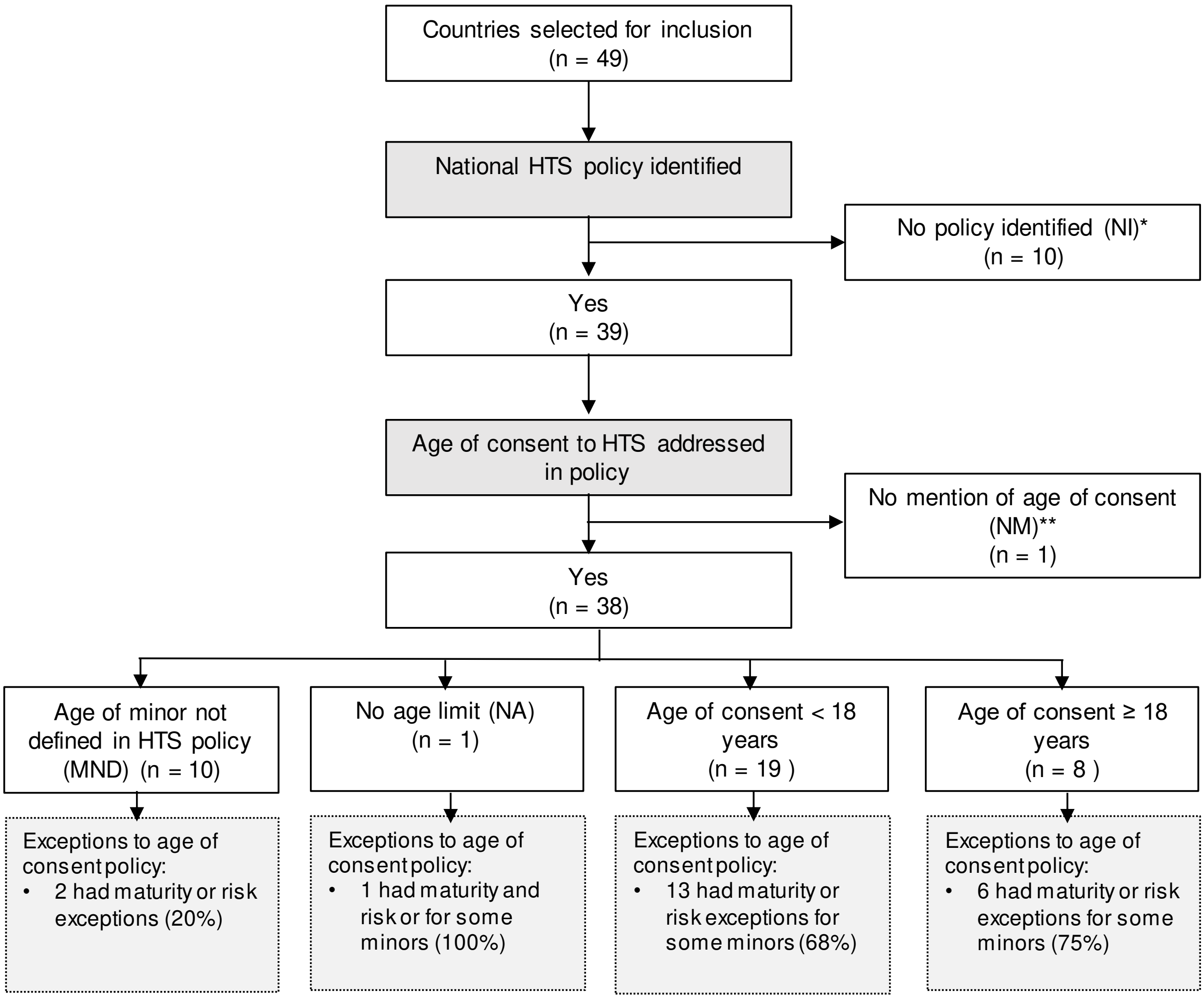

Supplement: Supplementary data [file bmjopen-2021-049673supp003.pdf]
