# Supplementary material for: Adolescents and age of consent to HIV testing: an updated review of national policies in sub-Saharan Africa
Source: BMJ Open. 2021 Sep 6;11(9):e049673. doi: 10.1136/bmjopen-2021-049673 (PMC8442095; doi:10.1136/bmjopen-2021-049673)
Supplement: Author's manuscript [file bmjopen-2021-049673.draft_revisions.pdf]

BMJ Open

BMJ Open is committed to open peer review. As part of this commitment we make the peer review history of every article we publish publicly available.

When an article is published we post the peer reviewers' comments and the authors' responses online. We also post the versions of the paper that were used during peer review. These are the versions that the peer review comments apply to.

The versions of the paper that follow are the versions that were submitted during the peer review process. They are not the versions of record or the final published versions. They should not be cited or distributed as the published version of this manuscript.

BMJ Open is an open access journal and the full, final, typeset and author-corrected version of record of the manuscript is available on our site with no access controls, subscription charges or pay-per-view fees (http://bmjopen.bmj.com).

If you have any questions on BMJ Open's open peer review process please email info.bmjopen@bmj.com

BMJ Open

Adolescents and age of consent to HIV testing: an updated review of national policies in sub-Saharan Africa

Journal:	*BMJ Open*
Manuscript ID	bmjopen-2021-049673
Article Type:	Original research
Date Submitted by the Author:	25-Mar-2021

Complete List of Authors:	Barr-DiChiara, Magdalena; World Health Organization<br>Tembo, Mandikudza ; London School of Hygiene and Tropical Medicine; Biomedical Research and Training Institute<br>Harrison, Lisa; Public Health England, South West Screening and Immunisations<br>Quinn, Caitlin; World Health Organization<br>Ameyan, Wole ; World Health Organization<br>Sabin, Keith; UNAIDS<br>Jani, Bhavin; World Health Organization<br>Jamil, Muhammad; World Health Organization<br>Baggaley, R; World Health Organization<br>Johnson, Cheryl; World Health Organization

Keywords:	Health policy < HEALTH SERVICES ADMINISTRATION & MANAGEMENT, International health services < HEALTH SERVICES ADMINISTRATION & MANAGEMENT, HIV & AIDS < INFECTIOUS DISEASES, PAEDIATRICS

SCHOLARONE™
Manuscripts

I, the Submitting Author has the right to grant and does grant on behalf of all authors of the Work (as defined in the below author licence), an exclusive licence and/or a non-exclusive licence for contributions from authors who are: i) UK Crown employees; ii) where BMJ has agreed a CC-BY licence shall apply, and/or iii) in accordance with the terms applicable for US Federal Government officers or employees acting as part of their official duties; on a worldwide, perpetual, irrevocable, royalty-free basis to BMJ Publishing Group Ltd ("BMJ") its licensees and where the relevant Journal is co-owned by BMJ to the co-owners of the Journal, to publish the Work in this journal and any other BMJ products and to exploit all rights, as set out in our licence.

The Submitting Author accepts and understands that any supply made under these terms is made by BMJ to the Submitting Author unless you are acting as an employee on behalf of your employer or a postgraduate student of an affiliated institution which is paying any applicable article publishing charge ("APC") for Open Access articles. Where the Submitting Author wishes to make the Work available on an Open Access basis (and intends to pay the relevant APC), the terms of reuse of such Open Access shall be governed by a Creative Commons licence – details of these licences and which Creative Commons licence will apply to this Work are set out in our licence referred to above.

Other than as permitted in any relevant BMJ Author's Self Archiving Policies, I confirm this Work has not been accepted for publication elsewhere, is not being considered for publication elsewhere and does not duplicate material already published. I confirm all authors consent to publication of this Work and authorise the granting of this licence.

For peer review only

Adolescents and age of consent to HIV testing: an updated review of national policies in sub-Saharan Africa

Magdalena Barr-DiChiara[1§], Mandikudza Tembo[2,3], Lisa Harrison[4], Caitlin Quinn[1], Wole Ameyan[1], Keith Sabin[5], Bhavin Jani[6], Muhammad S. Jamil[1], Rachel Baggaley[1], Cheryl Johnson[1]

1 Global HIV, Hepatitis and STI Programmes, World Health Organization, Geneva, Switzerland

2 MRC Tropical Epidemiology Group, London School of Hygiene and Tropical Medicine, London, United Kingdom

3 Biomedical Research and Training Institute, Harare, Zimbabwe

4 Brook, Young people's sexual health and well-being charity, Liverpool, United Kingdom

5 UNAIDS, Geneva, Switzerland

6 World Health Organization, Dar es Salaam, United Republic of Tanzania

§ Corresponding author:

Madgalena Barr-DiChiara
Department of Global HIV, Hepatitis, and STI Programmes
World Health Organization
Avenue Appia 20
1211 Geneva, Switzerland
+41 079 924 9388
barrdichiaram@who.int

E-mail addresses of authors:

MD: m.dichiara@gmail.com

MT: mandikudza.tembo@lshtm.ac.uk

LH: lisaharrisonwk@gmail.com

WA: ameyanw@who.int

RB: baggaleyr@who.int

CQ: quinnc@who.int

MJ: mjamil@who.int

CJ: johnsonc@who.int

For peer review only

Abstract

Objectives

In sub-Saharan Africa (SSA) where HIV burden is highest, access to testing, a key entry point for prevention and treatment remains low for adolescents. Access may be hampered by polices requiring parental consent for adolescents (aged 10-19) to receive HIV testing services (HTS). In 2013, WHO recommended countries review policies on age of consent for HTS. Here we investigate country progress and policies on age of consent for HIV testing.

Primary and secondary outcome measures

We reviewed national HTS policies in force in SSA, published from 2005 through November 2019, to assess landscape changes since 2013. Data were extracted on age of consent for HTS including exceptions based on risk and maturity. Descriptive analyses of included national polices were disaggregated by eastern and southern Africa (ESA) and western and central Africa (WCA) sub-regions.

Results

Forty national policies were reviewed, 38 were included: 19/38 (50%) permitted HTS for adolescents between 12 and 16 years without parental consent. Of these, six allowed HTS ≥12 years, two at ≥13, two at ≥ 14, five at ≥15, four at ≥16. In ESA, 71% of countries (n=15/21) allowed those of ≤16 years to access HTS while only 24% (n=6/25) of WCA countries allowed the same. Maturity exceptions for those who met criteria including marriage, sexual activity, pregnancy, or key population status were identified in 18 policies. In 2019, 63% of policies with clear age-based criteria (n=19/30) allowed adolescents of 12-16 years to access HIV testing without parental consent, an increase from only 37% (n=14/38) in 2013.

Conclusions

While many countries in SSA have revised their HTS policies, many do not yet specify age of consent. Revision of SSA consent to HTS policies, particularly in WCA, remains a priority to achieve the 2025 goal of 95% of people with HIV knowing their status.

STRENGTHS AND LIMITATIONS OF THIS STUDY

- This study provides a comprehensive review of national age of consent to HIV testing policies in sub Saharan Africa.
- Despite a robust search approach, some policy documents may not have been identified.
- Due to varied document formats of, the reviewers employed a non-automatized data extraction that was complemented by using key words to maintain repeatability.
- This review included national policy documents but did not assess national laws which contribute to a potentially limiting operational context to better understand guidance which informs routine implementation.

For peer review only

BACKGROUND

In the past decade, substantial but unequal progress has been made toward the global "90-90-90" fast-track goals: an estimated 81% of people living with HIV (PLHIV) know their status, 82% of aware PLHIV are on antiretroviral therapy (ART) and 88% of PLHIV on ART are achieving viral suppression.[1] Despite successes, nearly 19% of the 38 million people living with HIV remain undiagnosed and there are an estimated 1.7 million new HIV infections annually.[2]

Globally, three-quarters (approximately 140,000) of the new 170,000 annual HIV infections among adolescents (aged 10-19 years) are in girls.[3] In sub-Saharan Africa (SSA), home to nearly 90% of all adolescents and young PLHIV, adolescent girls and young women (AGYW) aged 15-24 years make up one quarter (about 330,000) of the new HIV infections although they represent just 10% of the population.[2] The number of young people in SSA is rapidly increasing and it is estimated that there will be almost 500 million 15-24-year-olds in the region by 2055. Recent projections point to declines in HIV infections among young people in SSA but not enough to reach global targets for low HIV incidence by 2030.[2 4]

Despite the high burden and incidence, and growing population, access to and uptake of HIV testing services (HTS), a pathway to essential prevention and treatment, remains remarkably low among adolescents.[5 6] In eastern and southern Africa (ESA) only 27% of adolescent girls and 16% of adolescent boys aged 15-19 received an HIV test in the last year and lower uptake was observed in western and central Africa(WCA)[3]. Access may be hampered by polices requiring parental consent for adolescents to receive HIV testing services (HTS).[7-9]

Adolescence is a time of rapid physical, sexual and cognitive development. Health services for adolescents, including HTS, should be delivered through youth-friendly approaches that reflect their diverse and evolving needs.[10] Social determinants, such as wealth and gender inequality as well as access to health and education services, impact adolescent health behaviour and outcomes in the near term, as they transition into adulthood and throughout the life course.[11-14]

For peer review only

Particularly underserved are adolescents and young people from key populations: men who have sex with men, transgender people, young people who sell sex[1] (people 10–24 years of age, including children 10–17 years who are sexually exploited and adults of 18–24 years who are sex workers), people who inject drugs, and people in prisons or closed settings. Key populations, especially those of younger ages, are criminalized and face stigma and discrimination from community members and providers.[15-17]

Adolescents at high ongoing risk who do not know their HIV status can benefit from HTS, prevention or treatment but access to these services is limited by policies requiring authorization from a parent or guardian.[7-9 18] While parental support can have a positive effect on adolescent's sexual health decision-making, for many adolescents who fear disclosing their sexual and other risk behaviours to their parents, those who do not have positive parental relationships, or for those who live in child-headed households, parental consent requirements pose a major barrier to HTS.[19] Empowering adolescents to freely access HTS and to choose how they want to test, including to use HIV self-testing (HIVST), supports equitable service delivery and the right to access health services.[20-22] Earlier analysis of the relationship between HTS coverage of young people in SSA and age of consent policies showed that allowing free adolescent access was associated with testing coverage of 14.0 percentage points higher for girls and 6.9 percentage points for boys.[8] In 2013, WHO guidance on HIV and adolescents encouraged countries to consider revising parental consent requirements to reduce barriers to HIV testing services.[23] Since that time, WHO has recommended HIVST, a convenient and private option which has been demonstrated highly acceptable for adolescents in SSA.[24 25] Countries in SSA, have moved to rapidly introduce and scale-up differentiated HIVST approaches that link people to diagnosis, treatment and care. Recent findings show that a when given the option of facility-based HTS or HIVST, adolescents and young people choose HIVST over conventional modalities, and are more likely to test and share tests with peers and partners when using HIVST.[26 27]

---

[1] While young adults who sell sex are considered sex workers, multiple international conventions describe the participation of those under 18 years of age in selling sex as a contravention of human-rights law, and those who do so are considered sexually exploited.

United Nations. United Nations Convention on the Rights of the Child (Article 1). U.N. Doc. A/Res/44/25, 1989 (http://www.ohchr.org/EN/ProfessionalInterest/Pages/CRC.aspx

United Nations. United Nations protocol to prevent, suppress, and punish trafficking in persons, especially women and children. Supplementing the United Nations convention against transnational organized crime. Article 3 (a-d); 2001 (A/45/49, http://www.unodc.org/documents/treaties/UNTOC/Publications/TOC%20Convention/TOCebook-e.pdf

For peer review only

Here we review policies in force following the 2013 WHO guidelines to evaluate country progress and uptake of WHO guidance on age of consent for HIV testing, including the age of consent for accessing HIVST.[7 23 28]

METHODS

We carried out a search of national policy documents related to HTS through searching WHO repositories, governmental and non-governmental and non-governmental websites, and contacting WHO country contacts and regional experts through April 2018 and updated our database through August 2020 (see supplementary material, search strategy).[29] All national policy documents pertaining to HTS were included; national HIV guidelines and strategic plans covering HIV and integrated guidelines on the prevention, treatment and care of HIV. For inclusion in the review, the policy must have included adolescent consent to HIV testing and be published by a WHO member state in SSA, of which there were 49. The policy language was not restricted but in policies other than English extraction was of age of consent was prioritized. The date of publication was not restricted and when more than one policy was identified for a country, the most current publication was included. When necessary, we contacted government officials, regional technical advisors, and other key experts to gather and/or validate information.

Policy data were extracted using a standardized Excel based form (see supplementary material, policy data extraction tool). Information extracted included age consent to HIV testing and/or self-testing as well as exceptions, including but not limited to individual maturity, key population status, emancipation, parenthood, marital status, sexual activity, and year of publication. HIV burden for included countries was classified as high (≥5%) or low (<5%) and collected from the Global AIDS Monitoring tool.[1 18] In addition to the policy assessment by the review team, national age of consent to HTS was also obtained from the National Commitments and Policy Instrument (NCPI) database, a global survey of HIV laws and policies.[28] Policies in languages other than English were reviewed with the aid of the translation software, Google Translate.

Two authors (MBD and MT) extracted information from policies independently.

For peer review only - http://bmjopen.bmj.com/site/about/guidelines.xhtml

Key word searching for terms including adolescent, child, minor, parent, and consent, was used to identify content addressing age of consent with full text review completed on the sections identified through this process. Disagreements between reviewers were resolved by discussion and consultation with a senior author.

Countries were classified according to two sub-regions: eastern and southern Africa (ESA) and western and central Africa (WCA)[2]. Policy data were then categorized using previously developed definitions and categories (Table 1): no mention, no adolescent access without parental consent, some adolescents may access without parental consent, and no minimum age limit.[8]

We conducted a descriptive analysis, complementing 2019 policy data with results reported in NCPI. We assessed country policy changes since 2013, including variation in age of consent to HIV testing and maturity exceptions, by comparing our results to the previous WHO review on age of consent.[1 7]

Patient and public involvement

Patients and/or the public were not involved in the design, or conduct, or reporting of this research.

RESULTS

A total of 40 national policies dated from 2005 through 2019 were identified among SSA countries, for nine countries no policy was identified. Of these, 2/40 (<5%) did not mention (NM) consent for adolescents or minors. 38 policies described consent for HTS and maturity exceptions and were included in the analysis: 10 policies did not provide definition of a minor (MND), one did not use age-based criteria for consent, and 27 policies detailed age-based of consent criteria (see supplementary material, included policies). Among included policies, eight limited HTS access without parental consent to persons ≥18 years of age while 19 (50%) permitted HTS for adolescents < 18 years without parental consent. Of these, six allowed HTS ≥12 years, two at ≥13, two at ≥ 14, five at ≥15, four at ≥16 (Table 2).

[2] We present data in two sub regions, ESA and WCA, which closely align with UNAIDS regions. We include Djibouti and Somalia, which are not in these UNAIDS regions, We do not include Algeria, which is traditionally north Africa, though it sits in the WHO African region.

For peer review only - http://bmjopen.bmj.com/site/about/guidelines.xhtml

The minimum age of consent was 12 years, reported in six countries, most of which were high burden (Lesotho, Mozambique, Rwanda, South Africa, Eswatini, and Uganda) (Table 2).

Among high burden countries, 80% (n=8/10) allowed younger adolescents to access testing independently of parents/guardians (Botswana, NA; Eswatini,12; Lesotho, 12; Malawi, 13; Mozambique, 1; Namibia, 14; South Africa, 12; Uganda, 12) while 20% (n=2/10) limited independent consent to older adolescents (Zambia, 16; Zimbabwe 16), (Table 3).

Further, all high burden policies permitted adolescents of 16 and younger to access HTS without parental consent pointing to alignment with WHO 2013 guidance and movement toward accessible testing services for adolescents where HIV is a major public health threat. NCPI age of consent policy reporting complemented our findings; however, some instances of disagreement were identified (Table 4).

Maturity exceptions

Maturity exceptions that waive parental consent requirements and effectively lower the age of consent for some groups of minors were identified in more than half (n=20/38) of policies that address age of consent, including those policies that did not specify age-based definitions of the age of majority (Table 5).

The most common maturity exceptions were: pregnancy (n=15/38), demonstrated maturity and understanding of test (n= 13/38), marriage (n=11/38), parenthood (n=10/38), and being at risk of contracting HIV due to behaviours or population group (n=9/38). Only six countries (Liberia, Rwanda, Sierra Leone, Somalia, South Sudan, Zambia) include maturity exceptions for adolescents from key populations group, all related to people who sell sex. Only one country (Liberia) included a maturity exception for street children. One country (Botswana) does not use age to determine adolescent access to HIV testing. Instead, the policy emphasised the need for providers to assess client maturity and client risks.

When stratified by sub-region, we identified notable differences the age of consent policies between ESA (n=21 countries) and WCA (n=25 countries) subregions. In WCA, for 32% of countries (n=8/25), no HTS policy was identified (Congo, Equatorial Guinea, Gabon, Guinea-Bissau, Mauritania, Niger, São Tomé and the Príncipe, Seychelles, Togo). Whereas, in ESA only two countries (Seychelles and Djibouti) had no identified policy.

For peer review only

Of the 17 WCA countries for which a policy was identified, 70% (n=12/17) included clearly defined age of consent for HTS and 5 countries (Benin, Burkina Faso, Central African Republic, Mali, and Sierra Leone) required parental consent for adolescents under 18 of age to test. In ESA there were six countries with an age of consent of 12 years whereas none were identified in WCA.

Changes in age of consent policies for HIV testing over time

Thirty-one of the country policies included (Table 2) were published after the 2013 release of WHO guidance on HIV and adolescents which called on policy-makers to detail clear and specific age-based consent language permitting adolescent access to HTS.[23] In 2013, clear age-based criteria for consent to HTS were present in 70% (n=28/40) of the laws and polices reviewed, we identified a similar proportion of policies with clear age-based consent language in 2019, 73% (n=28/38).[7] We observed a decrease in the proportion of polices with age-based consent criteria which permitted adolescents of 16 years or younger to access HTS without parental consent: in 2013 50% (n=14/28) allowed access without parental consent to those of 16 years or younger, and in 2019 68% (n=19/28) allowed access without parental consent to those of 16 years or younger. While a majority of policies had been updated since 2013, reduction in age of consent to HTS occurred in just six policies; in three high burden countries (Eswatini, Mozambique and Namibia) and just four low burden countries (Cote d'Ivoire, Gambia, Rwanda, United Republic of Tanzania).

HIV self-testing

No policies in the 2013 review included information on age of consent for HIVST. The first WHO recommendation on HIVST was introduced in 2016. In 2019, nearly half of (44%, 17/38) national policies included self-testing and 53% of these (n=9/17) clearly defined age of consent for access to HIVST. Among HVST policies, nine clearly defined age of consent for HIVST (Cote d'Ivoire, 16; Eritrea, 18; Eswatini, 16; Ghana, 16; Mali, 18; Somalia; 18, South Africa, 12 with directly assisted HIVST; South Sudan, 18, Tanzania, 18). Nine polices (Benin, Cameroon, Democratic Republic of Congo, Kenya, Lesotho, Malawi, Nigeria, Zambia and Zimbabwe) addressed HIVST but did not include clear definition of age of consent and among these were two standalone HIVST policies (Zambia and Zimbabwe). HIVST policies were diverse in format, some were standalone while others integrated HIVST into broader testing service polices. Two policies (Kenya and South Africa) explicitly enabled self-testing, but only with direct assistance for those under the age of 18 years, thus distinguishing HIVST from conventional provider delivered options.

For peer review only - http://bmjopen.bmj.com/site/about/guidelines.xhtml

Eswatini and Tanzania and Somalia had a higher age of consent for HIVST (16 years for Eswatini, 18 years for Tanzania) than for provider delivered methods (12 years for Eswatini, 15 years for Tanzania and Somalia).

NCPI

NCPI, a mechanism for monitoring national laws and policies, captures age of consent regulations for HIV testing and other reproductive health services among other themes.[28 74] Our review findings complement the policy reporting in NCPI but do not capture consistent country-level information on age of consent to HIV testing (Table 3). The discrepancy of our findings with those reported by NCPI highlight challenges in aggregating policy information in surveys and research. Since NCPI includes age of consent policy information covering HTS, ART and sexual and reproductive health and rights (SRHR) services it provides regular regional intelligence and offers a platform to routinely review, validate and engage around age of consent policies.

DISCUSSION

Our review found that half of policies in SSA allowed adolescents aged 16 and younger to provide independent consent for HIV testing.

We found the minimum specified age of consent for HTS in any SSA country was 12 years with one country reporting no age- based criteria. In comparison, the minimum specified age of consent for HTS in western Europe and north America, as 14 years, with some countries reporting no age based criteria, and many countries not reporting.[28] There has been substantial progress in SSA since 2013 when WHO encouraged countries to review and adapt consent polices to make HIV testing accessible to adolescents, with 31 updated policies identified. Among these, seven lowered the age of consent.[23] Policy makers in SSA have moved to address HIVST but there is more work to be done in both developing policy and including clearly defined age of consent language. Our review also identified gaps in age of consent policies in SSA, particularly WCA: overall about a quarter of policies (n=10/38) did not clearly describe age of consent and 21% restricted HTS to those 18 years or above.

For peer review only

In this review we assessed only HIV testing policies. However, polices should facilitate integration and linkage to promote comprehensive adolescent health packages that deliver the benefits of HIV prevention, treatment, and other SRHR services so that public health impact can be realized. In countries with clear age of consent policies (including many in ESA), alignment of HTS policies with other SRHR and HIV prevention policies should be prioritized. In countries without polices, or in those with vague policies (including many in WCA) development of guidance with clear consent language that makes HIV testing, SRHR and HIV services broadly available to adolescents is needed. In all settings, key population and sexually active adolescents need policies that allow them to provide their own consent for HTS.[28] One recent country example of policy change took place in Tanzania, where in 2019 following a period of coordinated political advocacy, parliamentary support for lowering age of consent for HIV testing from 18 to 15 years was obtained; age of consent for HIV self-testing remained at 18 years of age.

A highlight of this review is the assessment of policies around HIVST, a highly acceptable way to engage adolescents in HIV testing and to make testing accessible for adolescents and young people.[75] In 2013, only two countries (Kenya and Unites States of America) had HIVST policies, and there were no WHO recommendations regarding their use. Further, the United States Food and Drug Administration restricted distribution by requiring proof of age for those accessing HIVST and limiting use to those of 17 years and older although initial studies included those of 14 years and older in sub-group analysis.[76]

Following the release of the first WHO guidance on self-testing for HIV in 2016, our 2019 review identified 17 country policies that addressed HIVST. Adolescents who self-test need tools and resources to support linkage to prevention and treatment services and approaches that deliver self-testing to this population through assisted and unassisted methods have been demonstrated effective and acceptable in SSA.[75] Two countries (South Africa and Kenya) stipulated assisted HIVST approaches for younger adolescents, thus making the modality available while ensuring a minimum standard of support for performing the self-test, interpreting the result, and linking to other services.

Policies frequently included maturity exceptions that eliminate parental consent requirements for adolescents seeking HTS, including those from key populations who are at particularly high risk of HIV and may also face social and structural barriers to testing, prevention and care services.[77]

For peer review only

Many other vulnerable adolescents are left out and overlooked: only six countries (Liberia, Rwanda, Sierra Leone, Somalia, South Sudan, Zambia) have policies that address adolescents who sell sex and exceptions for adolescents from other key populations were not identified. Exceptions did frequently address adolescent girls; those for marriage, pregnancy, involvement in sex work, and sexual activity may effectively lower age of consent for some girls, however, girls may not feel empowered to seek health services or to take a test when services are not designed to be accessible to adolescents. Overall, we found great variation in maturity exceptions and are not able to ascertain how these are interpreted and put into practices by implementers and service providers or how they ultimately impact access to HIV testing for adolescents. In settings where maturity exception policies are present, programmes should assess fidelity as well as seek and consider adolescent perspectives while they aim to strengthen service delivery for HIV testing and prevention.

Strengths and limitations

Documents from nearly forty countries were reviewed using a standard approach to extraction and analysis. Despite a robust approach, some policy documents may not have been identified. Due to varied document formats of, the reviewers employed a non-automatized data extraction that was complemented by using key words to maintain repeatability. This review included national policy documents but did not assess national laws which contribute to a potentially limiting operational context to better understand guidance which informs routine implementation.[9] Since the review did not assess implementation, it does not provide insight into how policies ultimately impact HIV testing service delivery and access for adolescents.

Further policy reviews and national assessments that address alignment on age of consent within and between HIV testing, prevention and treatment, STI and other sexual and reproductive health services will illuminate opportunities to strengthen policy and implementation. Finally, while a significant proportion of policies reviewed did not define the age of consent which limited assessment of regional trends, this did not hinder our ability to assess whether national policies contained clear description of age of consent to test.

For peer review only

CONCLUSIONS

Since 2013, 31 SSA countries have updated policies that include age of consent to HTS, with seven reducing the age of consent to 12 years or eliminating age-based criteria. Our findings suggest that adolescents in 19/38 (50%) of SSA countries may access HTS without consent of a parent or guardian at varying ages: 16, 15, 14, or 12 years. Access to HTS for adolescents remains limited in WCA, where policies are fewer, older, and have vague or no age of consent language. HTS polices should acknowledge the right of adolescents to provide their own consent to testing, particularly through newly introduced approaches like HIVST, and be aligned with national SRHR guidance so that adolescents can receive the benefits of services for contraception, voluntary medical male circumcision, sexually transmitted infections, HIV prevention and antiretroviral treatment. Whilst polices may acknowledge that where adolescents have supportive parents or trusted older relatives, involving these in testing is often of great benefit to coping with a result and discussing linkage to other services post testing. As countries renew efforts to reach the first 95 and improve outcomes for adolescents, lowering age of consent for HIV testing should continue to be prioritized.

ORCiD: Magdalena Barr-DiChiara https://orcid.org/0000-0002-7404-7843

List of abbreviations

AGYW: adolescent girls and young women

ART: antiretroviral therapy

ESA: eastern and southern Africa

HIVST: HIV self-testing

HTS: HIV testing services

MME: mature minor exceptions

MND: minor not defined

NA: no minimum age limit

NCPI: National Commitments and Policy Instrument

NM: no mention

PCR: parental consent required

PLHIV: people living with HIV

SAC: some adolescents may access without parental consent

For peer review only

DECLARATIONS

Ethics approval Not applicable.

Availability of data and materials Data may be obtained from a third party and some data are publicly available. Most policies included in this review are available through the following websites: (1) https://aidsfree.usaid.gov/resources/guidancedata/hts. (2) http://www.hivpolicywatch.org/database.html. If information on a policy cannot be found through these sources, please contact the authors of this review for additional information.

Competing interests The authors declare no competing interests. The contents in this article are those of the authors and do not necessarily reflect the view of the World Health Organization.

Funding Funding for this review was provided by the U.S. President's Emergency Plan for AIDS Relief (PEPFAR) through World Health Organization and USAID's Consolidated WHO Grant, Agreements US-2015-0839 and US-2016-940.

Patient consent for publication Not required.

Not commissioned; externally peer reviewed.

Contributors CJ and RB devised the review. MBD and CJ designed the protocol. MBD conducted the search, and MT conducted the screening and data extraction. CJ and RB supervised the review.

MBD and MT qualitatively synthesized the findings and conducted descriptive analyses. MBD and LH drafted the manuscript with input from MT, CJ and MJ. All authors reviewed the draft, provided critical review, and read and approved the final manuscript.

Acknowledgements The authors thank all country and regional contacts for policies for the review.

For peer review only

REFERENCES

1. UNAIDS. Global factsheet 2019. Geneva: United Nations Joint Programme on HIV/AIDS, 2020. Available: https://aidsinfo.unaids.org/# Acessed 21 Dec 2020.
2. UNAIDS. Siezing the moment, tackling entrenched inequalities to end epidemics: global AIDS update 2020. Geneva: United Nations Join Programme on HIV/AIDS, 2020. Available: https://aids2020.unaids.org/report/ Accessed 7 Jul 2020.
3. UNICEF. UNICEF Data: Adolescent HIV prevention https://data.unicef.org/topic/hivaids/adolescents-young-people/: UNICEF; 2020 Available: https://data.unicef.org/topic/hivaids/adolescents-young-people/ Accessed: 7 Jul 2020.
4. UNAIDS. Progress towards the start free, stay free, AIDS free targets. Geneva: United Nations Joint Programme on HIV/AIDS, 2020. Avaialble: https://www.unaids.org/sites/default/files/media_asset/start-free-stay-free-aids-free-2020-progress-report_en.pdf Accessed: 7 Jul 2020.
5. Wong V, Murray K, Phelps B, et al. Adolescents, young people, and the 90-90-90 goals: a call to improve HIV testing and linkage to treatment. *AIDS* 2017;31(Supplement 3):S191–S94. doi: https://doi.org/10.1097/QAD.0000000000001539
6. Kurth A, Lally M, Choko A, et al. HIV testing and linkage to services for youth. *J Int AIDS Soc* 2015;18(Suppl 1):19433. doi: https://doi.org/10.7448/IAS.18.2.19433
7. WHO. HIV and adolescents: guidance for HIV testing and counselling and care for adolescents living with HIV: annex 15: Adolescent consent to testing: a review of current policies and issues in sub-Saharan Africa. Geneva: World Health Organization, 2013. Available: https://apps.who.int/iris/handle/10665/95147 Accessed: 7 July 2020.
8. McKinnon B, Vandermorris A. National age-of-consent laws and adolescent HIV testing in sub-Saharan Africa: a propensity-score matched study. *Bull World Health* 2019;97(1):42–50. doi: https://doi.org/10.2471/BLT.18.212993
9. Eba P, Lim H. Reviewing independent access to HIV testing, counselling and treatment for adolescents in HIV-specific laws in sub-Saharan Africa: implications for the HIV response. *J Int AIDS Soc* 2017;20(1): 21456. doi: https://doi.org/10.7448/IAS.20.1.21456
10. WHO. WHO recommendations on adolescent sexual and reproductive health and rights. Geneva: World Health Organization, 2018. Available: https://www.who.int/publications/i/item/working-to-safeguard-the-health-well-being-and-human-rights-of-adolescents-with-who-guidelines Accessed: 7 July 2020
11. Viner R, Ozer E, Denny S, et al. Adolescence and the social determinants of health. *Lancet* 2012;379(9826):1641-52. doi: doi: 10.1016/S0140-6736(12)60149-4
12. Stoner M, Nguyen N, Kilburn K, et al. Age-disparate partnerships and incident HIV infection in adolescent girls and young women in rural South Africa. *AIDS* 2019;33(1):83–91. doi: https://doi.org/10.1097/QAD.0000000000002037
13. Brown K, Williams DB, Kinchen S, et al. Status of HIV Epidemic Control Among Adolescent Girls and Young Women Aged 15–24 Years — Seven African Countries. MMWR 2015–2017. 2018; 67(1):29–32.
14. Starrs A, Ezeh A, Barker G, et al. Accelerate progress—sexual and reproductive health and rights for all: report of the Guttmacher–Lancet Commission. *Lancet* 2018;391 doi: http://dx.doi.org/10.1016/
15. Baggaley R, Armstrong A, Dodd Z, et al. Young key populations and HIV: a special emphasis and consideration in the new WHO Consolidated Guidelines on HIV Prevention, Diagnosis, Treatment and Care for Key Populations. *J Int AIDS Soc* 2015;18(2 S1)
16. Cornell M, Dovel K. Reaching key adolescent populations. *Curr Opin HIV AIDS* 2018;13(3):274–80. doi: https://doi.org/10.1097/COH.0000000000000457
17. WHO. HIV and young people who sell sex. Geneva: World Health Organization 2015. Available: https://www.unaids.org/sites/default/files/media_asset/2015_young_people_who_sell_sex_en.pdf Accessed: 10 Dec 2020.
18. WHO. Consolidated guidelines on HIV testing services, 2019 Geneva: World Health Organization, 2019. Available: https://www.who.int/publications/i/item/978-92-4-155058-1 Accessed: 21 Dec 2020.
19. Sam-Agudu N, Folayan M, Ezeanolue E. Seeking wider access to HIV testing for adolescents in sub-Saharan Africa. *Pediatr Res* 2016;79(6):838–45. doi: doi: 10.1038/pr.2016.28
20. WHO. The voices, values and preference of adolescents on HIV testing and counselling: consultation for the development of the World Health Organization HIV testing and counselling guidelines for adolescents. Geneva: World Health Organization, Royal Tropical Institute, 2013. Available: https://apps.who.int/iris/handle/10665/95143 Accessed: 7 Jul 2020.

For peer review only

21. Convention on the Rights of the Child. 1989. Available: https://www.ohchr.org/en/professionalinterest/pages/crc.aspx Accessed: 7 Jul 2020
22. General comment No. 4: Adolescent Health and Development in the Context of the Convention on the Rights of the Child: UN Committee on the Rights of the Child (CRC), 2003.
23. WHO. HIV and adolescents: guidance for HIV testing and counselling and care for adolescents living with HIV. Geneva: World Health Organization, 2013.
24. WHO. Guideline on HIV self-testing and partner notification: Supplement to consolidated guidelines on HIV testing services. Geneva: World Health Organization, 2016. Available: https://www.who.int/hiv/pub/vct/hiv-self-testing-guidelines/en/ Accessed: 7 Jul 2020.
25. Hatzold K, Gudukeya S, Mutseta M, et al. HIV self-testing: breaking the barriers to uptake of testing among men and adolescents in sub-Saharan Africa, experiences from STAR demonstration projects in Malawi, Zambia and Zimbabwe. *J Int AIDS Soc* 2019;22(S1) doi: https://doi.org/10.1002/jia2.25244
26. Pettifor A, Lippman S, Kimaru L, et al. HIV self-testing among young women in rural South Africa: A randomized controlled trial comparing clinic-based HIV testing to the choice of either clinic testing or HIV self-testing with secondary distribution to peers and partners. *EClinical Medicine* 2020 doi: https://doi.org/10.1016/j.eclinm.2020.100327
27. Indravudh P, Sibanda E, d'Elbée M. 'I will choose when to test, where I want to test': investigating young people's preferences for HIV self-testing in Malawi and Zimbabwe. *AIDS* 2017;31(Suppl 3):S2013-S212. doi: doi: 10.1097/QAD.0000000000001516.
28. UNAIDS. UNAIDS Laws and Policies Analytics. United Nations Join Programme on HIV/AIDS, 2020. Available: http://lawsandpolicies.unaids.org/about Accessed: 7 Jul 2020.
29. Fonner V, Sands A, Figueroa C, et al. Country adherence to WHO recommendations to improve the quality of HIV diagnosis: a global policy review. *BMJ Glob. Health* 2020;5:e001939. doi: http://dx.doi.org/10.1136/bmjgh-2019-001939
30. Normas de Tratamento Antirretroviral. República De Angola: Ministerio da Saude, 2015.
31. Directives Nationales de Traitment Anti Retrovirale de L'Infection par au Burundi: Ministre de la Sante Publique et de la Lutte Contre le SIDA 2014.
32. National Guideline on the Prevention and Management of HIV in Cameroon. Government of the Republic of Cameroon: Ministry of Public Health, 2015.
33. Procedimentos, normas e direcrizes do atendimento das pessoas Cabo Verde: Direcao-Geral de Saude, Programa de Luta Contra a SIDA/IST, Tuberculose e Lepra, 2005.
34. Manuel de Formation en Counseling VIH/SIDA/IST. Republic du Tchad: Ministere de la Sante Publique, 2011.
35. Guide de prise en charge de l'infection à VIH Aux Comores. Union des Comores: Vice Présidence en charge du Ministère de la Santé , de la Solidarité 2007.
36. Guide de Prise en Charge Integree du VIH en Republique Democratique du Congo. Gouvernement de la République démocratique du Congo: Ministere de la Sante Publique, 2017.
37. Cadre strategique national de lutte contre le SIDA 2018-2022. Conakry, Guinea Comite National de Lutte Contre le SIDA, Secretariat Executif, , 2018.
38. National Guidelines for HIV Prevention Treatment and Care. Government of Nigeria: Federal Ministry of Health, 2016.
39. National Consolidated HIV Testing Services Guidelines. Government of Sudan: Federal Ministry of Health, 2016.
40. Handbook of the Botswana 2016 Integrated HIV Clinical Care Guidelines. Government of the Republic of Botswana: Ministry of Health, 2016.
41. National Guidelines on the Use of Antiretroviral Therapy for HIV Prevention and Treatment. Government of Lesotho: Ministry of Health, 2016.
42. Directriz nacional para a implementação do aconselhamento e testagem em saúde: República de Moçambique, Ministério da Saúde, Direcção Nacional de Assistência Médica, PNC ITS/HIV/SIDA, 2015.
43. National Guidelines for Prevention and Management of HIV and STIs. Government of the Republic of Rwanda: Ministry of Health, 2016.
44. National HIV Testing services: Policy. Government of the Republic of South Africa: Department of Health, 2016.
45. Swaziland Integrated HIV Management Guidelines. Government of the Kingdom of eSwatini Ministry of Health, 2018.
46. Consolidated Guidelines for Prevention and Treatment of HIV in Uganda Ministry of Health, Republic of Uganda, 2016.
47. National Policy Guidelines on HIV AND AIDS. Government of Gambia: National AIDS Council, 2014.
48. Malawi HIV Tetsing Services Guidelines. Government of Malawi: Ministry of Health, 2016.

For peer review only

49. Integrated Guidelines for Prevention, Testing, Care and Treatment of HIV and AIDS in Liberia. Republic of Liberia: Ministry of Liberia, 2015.
50. National Guidelines for Antiretroviral Therapy. Government of the Republic of Namibia Ministry of Health and Social Services, 2016.
51. National Guidelines for Comprehensive HIV Prevention, Care and Treatment: Federal Ministry of Health, Ethiopia, 2017.
52. Kenya HIV Testing Services Guidelines: National AIDS Control Programme 2015.
53. Politique, Normes et Protocoles des Services Depistage du VIH: Republique du Sénégal Ministere de la Sante Publique, 2017.
54. Somalia HIV Testing and Counseling Policy: Ministry of Health, 2017.
55. UNAIDS. United Republic of Tanzania lowers age of consent for HIV testing Geneva: United Nations Joint Programme on HIV/AIDS; 2019 [Available from: https://www.unaids.org/en/resources/presscentre/featurestories/2019/november/united-republic-of-tanzania-lowers-age-of-consent-for-hiv-testing accessed 7 July 2020.
56. Directives technique de prevention du sida et de pris en charge de personnes vivant avec le VIH. Abidjan: Ministere de la sante et de l'hygiene, 2019.
57. Differentiated service delivery for HIV in Ghana: an operational manual National AIDS Control Programme, 2017.
58. HIV Testing Services National Guidelines: Ministry of Health, Republic of Zambia, 2016.
59. Zimbabwe National Guidelines on HIV Testing and Counselling, second edtion. In: Care MoHaC, ed., 2014.
60. Strategie Nationale de Demedicalisation du Depitage du VIH au Benin, 2017.
61. Normes et Directives Nationales de Conseil Depistage de L'infection a VIH au Burkina Faso. Gouvernement de Burkina Faso: Ministere de la Sante, 2008.
62. Guide du Depistage et Conseil du VIH la L'initiative du Prestataire. Gouvernement de la Republic Centrafricaine Ministere de la Sante Publique, 2010.
63. Eritrea National Guidelines for HIV Testing Services. Asmara: Ministry of Health, Deptartment of Public Health, Communicable Diseases Control Division, National AIDS Control Program, 2019.
64. Counseling et Depistage du VIH a Madagasca Rnormes et Procedures. Republic of Madagascar: Le Ministre de la Santé Publique, 2011.
65. Normes et Procedures des Services de Despistage du VIH au Mali: Haut Conseil National de Lutte Contre le SIDA, 2017.
66. Guidelines for HIV Tetsing Services in Sierra Leone National AIDS Control Programme: Ministry of Health and Sanitation, 2017.
67. Consolidated Clinical Guidelines on Use of Antiretroviral Drugs for HIV Treatment and Prevention Government of the Republic of South Sudan: Ministry of Health, 2017.
68. Directives Nationales D'utilisation des Antiretroviraux Pour la Prevention et le Traitement du VIH. Gouvernement de la Republique du Burundi: Ministere de la Sante, 2016.
69. Document de Politique,Normes et Procedures des Services de Depistage du VIH en Côte D'ivoire. Le gouvernement de Cote D'Ivoire: Ministère en charge de la Santé et de l'Hygiene Publique, 2016.
70. National HIV and AIDS Policy: Republic of Mauritius, 2010.
71. National Guidelines for the Management of HIV and AIDS. Government of the United Republic of Tanzania: Ministry of Health, Community Development, Gender, Elderly, and Children 2017.
72. Lesotho National Policy & Guidelines on HIV Testing Services: Ministry of Health Lesotho, 2017.
73. Guidelines for Antiretroviral Therapy for the Prevention and Treatment of HIV in Zimbabwe, 2016: Ministry of Health and Child Care, 2016.
74. Torres M, Gruskin S, Buse K, et al. Monitoring HIV-Related Laws and Policies: Lessons for AIDS and Global Health in Agenda 2030. *AIDS Behavior* 2017;21:S51–S61. doi: https://doi.org/10.1007/s10461-016-1621-5
75. Jamil M, Eshun-Wilson I, Charles Witzel T*ea*. Effectiveness of HIVST Distribution Models in the General Population in SSA: A Systematic Review [abstract]. International Conference on AIDS and STIs in Africa. Kigali, , 2019.
76. OraQuick® in-home HIV test summary of safety and effectiveness. Washington, DC: United States Food and Drug Administration, 2012.
77. Staveteig S, Croft T, Kampa K, et al. Reaching the 'first 90': Gaps in coverage of HIV testing among people living with HIV in 16 African countries. *PloS one* 2017;12(10):e0186316. doi: https://doi.org/10.1371/journal.pone.0186316

For peer review only

Table 1. Definitions for country policies to age of consent to HIV testing

**Policy category**	**Definition**
No mention (NM)	Policy does not mention adolescents or minors or age of consent for HTS.
Minor not defined (MND)	Age of minor not defined.
Parental consent required (PCR)	Age of consent to HIV testing is 18 years or above. Ability to consent is defined by age.
Some adolescents may access without parental consent (SAC)	Age of consent to HIV testing is below 18 years. Minimum age to consent for HTS varies by country. Ability to consent is defined by age.
No minimum age limit (NA)	There is no minimum age limit for independent consent to HIV testing.
Mature minor exceptions (MME)	Minors classified as mature may access to HIV testing without parental consent. Criteria for exceptions to minimum age of consent may include:<br>• Shows maturity and understanding of the process and potential results (health-care provider discretion)<br>• At risk of contracting HIV or sexually active<br>• Symptomatic<br>• Pregnant<br>• Parent (adolescent is already a parent)<br>• Head of household<br>• Married<br>• Child engaged in commercial sex work<br>• Street child<br>Mature minor exceptions are not mutually exclusive of other approaches including: no adolescent access, some adolescent access, or no age limit.

For peer review only

Table 2. Age of consent to HTS policies in sub-Saharan Africa

**National policies reviewed (n=49) [categories not mutually exclusive]**	**No. of countries (%)**
Policy existing for HTS, age of consent clearly defined (including no age limit)	30 (61.2%)
Policy existing for HTS, but no age of consent specified*	8 (16.3%)
Total number of countries with policies discussing age of consent to HTS	*38 (77.6%)*
Policy existing for HTS, but no mention of 'adolescent' or 'minor'	1 (2.0%)
No policy for HTS	10 (20.4%)
Total number of countries either without HTS policies, or that have policies that do not mention adolescents or minors	*11 (22.4%)*
**National age of consent to HTS (n=38)**<br>The year following the country indicates the year of publication for national policy.	**No. of countries (%)**
**Age of minor not defined**<br>Angola, 2015[30]; Burundi, 2014[31]; Cameroon, 2015[32]; Cape Verde, 2005[33]; Chad, 2011[34]; Comoros, 2007[35]; DRC, 2017[36]; Guinea, 2018[37]; Nigeria, 2016[38]; Sudan, 2016[39]	10 (26.3%)
**No age limit – all or most adolescents eligible****<br>Botswana, 2016[40]	1 (2.6%%)
**12 years**<br>Lesotho 2016[41]; Mozambique, 2015[42]; Rwanda, 2016[43]; South Africa, 2016[44]; Eswatini, 2018[45]; Uganda, 2016[46]	6 (15.8%%)
**13 years**<br>Gambia, 2014[47]; Malawi, 2016[48]	2 (5.3%%)
**14 years**<br>Liberia, 2015[49]; Namibia, 2016[50]	2 (5.3%)
**15 years**<br>Ethiopia, 2017[51]; Kenya 2015[52]; Senegal, 2017[53]; Somalia, 2017[54]; United Republic of Tanzania, 2019[55]	5 (13.2%)
**16 years**<br>Côte d'Ivoire, 2019[56]; Ghana, 2017[57]; Zambia, 2016[58]; Zimbabwe, 2014[59]	4 (10.5%)
**18 years**<br>Benin, 2017[60]; Burkina Faso, 2008[61]; Central African Republic, 2010, [62]; Eritrea, 2019[63]; Madagascar 2011[64]; Mali, 2017[65]; Sierra Leone, 2017[66]; South Sudan 2017[67]	8 (21.1%)

* These countries used the term 'minor' in their guidance, but do not specify an age.
** These countries stipulate considerations other than age to allow access to HTS (e.g. shows maturity, of reproductive age, or married, pregnant, or engaged in behaviour that would put them at risk).

For peer review only

Table 3. HIV testing policies identified, and age of consent to HTS in sub-Saharan Africa

	Country	Age of consent to HTS	Sub-region	HIV burden*
1	Angola	MND[30]	ESA	Low
2	Benin	18 (Age 15 if adolescent shows maturity)[60]	WCA	Low
3	Botswana	NA[40]	ESA	High
4	Burkina Faso	18 (MME: from 15 years of age.)[61]	WCA	Low
5	Burundi	MND[68]	WCA	Low
6	Cameroon	MND (MME: HTS from age 10-13 with parental consent, HTS from age 14-19 if sexually active or family head)[32]	WCA	Low
7	Cape Verde	MND [33]	WCA	Low
8	Central African Republic (the)	18 (MME: if there is no parent or guardian and a child is able to manage the decision, a minor may access HTS independently)[62]	WCA	Low
9	Chad	MND[34]	WCA	Low
10	Comoros (the)	MND[35]	ESA	Low
11	Congo (the)	NI	WCA	Low
12	Côte d'Ivoire	16[69]	WCA	Low
13	Democratic Republic of the Congo	MND[36]	WCA	Low
14	Djibouti	NI	ESA	Low
15	Equatorial Guinea	NI	WCA	High
16	Eritrea	18[63]	ESA	Low
17	Eswatini	12 (MME: people of 16 years or more may access HIVST)[45]	ESA	High
18	Ethiopia	15 (MME: mature minors of 13-15 years may consent to HIV testing, those less than 15 years of age cannot consent to HIV testing) [51]	ESA	Low
19	Gabon	NI	WCA	Low
20	Gambia (the)	13 (MME: for those below age 13, parental consent is required unless it is an emergency)[47]	WCA	Low
21	Ghana	16 (MME: any person who is between 14 and 16 years and is sexually active, married, pregnant, a parent or requests HTS is considered to be a mature minor and is able to give full informed consent)[57]	WCA	Low
22	Guinea	MND[37]	WCA	Low
23	Guinea-Bissau	NI	WCA	Low
24	Kenya	15 (MME: emancipated minor exception, survivors of sexual violence who are under the age of 15 may access HIV testing without parental consent)[52]	ESA	Low
25	Lesotho	12[41]	ESA	High
26	Liberia	14 (MME: any child who is married, pregnant, works as a commercial sex worker, is a street teenager, family head or with a history of sexual intercourse is regarded as a mature or emancipated minor and can consent for HIV testing)[49]	WCA	Low
27	Madagascar	18[64]	ESA	Low

For peer review only

	Country	Age of consent to HTS	Sub-region	HIV burden*
28	Malawi	13 (MME: minors of fewer than 13 years of age may consent to HTS)[48]	ESA	High
29	Mali	18[65]	WCA	Low
30	Mauritania	NI	WCA	Low
31	Mauritius	NM[70]	ESA	Low
32	Mozambique	12 (MME: adolescents married, living as if married, sexually active, head of household may give consent for HIV testing)[42]	ESA	High
33	Namibia	14[50]	ESA	High
34	Niger (the)	NI	WCA	Low
35	Nigeria	MND [38]	WCA	Low
36	Rwanda	12[43]	ESA	Low
37	São Tomé and the Príncipe	NI	WCA	Low
38	Senegal	15[53]	WCA	Low
39	Seychelles	NI	ESA	Low
40	Sierra Leone	18 (MME: child survivors of sexual assault or abuse, married young people under age 18, pregnant, parents, minors engaged in behaviour which puts them at risk, child sex workers)[66]	WCA	Low
41	Somalia	15 (MME)[54]	ESA	Low
42	South Africa	12 (For HIVST, children between age 12 and 18 years should use directly assisted testing options)[44]	ESA	High
43	South Sudan	18 (MME: married, pregnant, parents, emancipated)[67]	ESA	Low
44	Sudan	MND (MME: adolescent KP)[39]	ESA	Low
45	Togo	NI	WCA	Low
46	Uganda	12[46]	ESA	High
47	United Republic of Tanzania (the)	15[55 71]	ESA	Low
48	Zambia	16[58]	ESA	High
49	Zimbabwe	16 (MME: shows maturity, at risk of HIV, symptomatic, pregnant)[59]	ESA	High

MND: Minor not defined
NA: No age of consent to HTS described in policy
NI: No information
NL: No age of consent to sex law

*Countries were classified as low- and high- HIV burden using an HIV prevalence threshold of 5%. Data was obtained from Global AIDS Monitoring, 2020 [1].

For peer review only

Table 4. Age of consent to HTS in sub-Saharan Africa national policies, 2013, 2019, and the National Commitments and Policy Instrument (NCPI)

Age of consent policy	WHO 2013* (n = 33)		WHO 2019** (n = 38)		NCPI*** (n= 49)	
	Name of countries	No. of countries (%)	Name of countries	No. of countries (%)	Name of countries	No. of countries (%)
No information/No policy identified, (NCPI: no data)	Chad, The Comoros, Equatorial Guinea, Eritrea, Gabon, The Gambia, São Tomé and the Príncipe, South Sudan	8	Congo, Djibouti, Equatorial Guinea, Gabon, Guinea-Bissau, Mauritania, Niger, Sao Tome and Principe, Seychelles, Togo	10	Cabo Verde, Cameroon, Chad, Comoros, Eritrea Guinea-Bissau, Sao Tome and Principe, Somalia	7 (14.2%)
Policy existing for HTS, but no mention of 'adolescent' or 'minor'	Mauritania	1 (2.1%)	Mauritius[70]	1 (2.6%)	-	-
Age of minor not defined	Angola, Benin, Burundi, Guinea-Bissau, Madagascar, Mali, Togo	7 (17.5%)	Angola[30], Burundi[31], Cameroon[32]¸ Cape Verde, Chad[34], Comoros[35], DRC[36], Guinea[37], Nigeria[38], Sudan[39]	10 (26.3%)	-	-
No age limit – all or most adolescents eligible	Botswana, Cape Verde, Kenya, Mauritius, Somalia (Somaliland)	5 (12.5%)	Botswana[40]	1 (2.6%)	-	-
12 years (NCPI: yes, for adolescents younger than 12 years)	Lesotho, South Africa, Uganda	3 (7.5%)	Lesotho[72], Mozambique[42], Rwanda[43], South Africa[44], Eswatini[45], Uganda[46]	6 (15.8%)	Rwanda	1 (2.0%)
13 years	Malawi	1 (2.5%)	Gambia[47], Malawi[48]	2 (5.3%)	-	0
14 years (NCPI: yes, for adolescents younger than 14 years)	Liberia	1 (2.5%)	Liberia[49], Namibia[50]	2 (5.3%)	Benin, Central African Republic, Congo, Gabon, Gambia, Lesotho, Liberia, Malawi, Mozambique, Namibia, Senegal, South Africa, Togo, Uganda	15 30.6%)
15 years	Ethiopia, Rwanda, Senegal	3 (7.5%)	Ethiopia[51], Kenya[52], Senegal[53], Somalia[54], United Republic of Tanzania[55]	5 (13.2%)	-	0
16 years (NCPI: yes, for adolescents younger than 16 years)	Congo, Mozambique, Namibia, Swaziland, Zambia, Zimbabwe	6 (15.0%)	Côte d'Ivoire[69], Ghana[57], Zambia[58], Zimbabwe[59]	4 (10.5%)	Botswana, Burundi, Cote d'Ivoire, Djibouti, Equatorial Guinea, Guinea, Niger, Zambia, Zimbabwe	9 (18.3%)

For peer review only

Age of consent policy	WHO 2013* (n = 33)		WHO 2019** (n = 38)		NCPI*** (n= 49)	
18 years<br>(NCPI: yes for adolescents less than 18 years)	Burkina Faso, Cameroon, Central African Republic, Côte d'Ivoire, Democratic Republic of the Congo, Djibouti, Ghana, Guinea, Niger, Nigeria, Seychelles, Sierra Leone, Sudan, United Republic of Tanzania	14 (35.0%)	Benin[60], Burkina Faso[61], Central African Republic[62], Eritrea[63], Madagascar[64], Mali[65], Sierra Leone[66], South Sudan[67]	8 (21.1%)	Angola, Burkina Faso, Democratic Republic of Congo, Eswatini, Ethiopia, Ghana, Kenya, Madagascar, Mali, Mauritius, Nigeria, Seychelles, Sierra Leone, South Sudan, Sudan, United Republic of Tanzania	17(35.6%)

*2013 review included both policies and laws while this review included only policies [7]

**Findings identified in the context of this policy review

***Data reported in the UNAIDS National Commitments and Policy Instrument, 2017, 2018, 2019

For peer review only

For peer review only

Table 5. Exceptions for HTS below age of consent

**Exceptions for testing below stated legal age of consent**<br>The age in parentheses indicates the national age of consent to HTS before the exception is applied.	**No. of countries**
**Shows maturity and understanding of the process and potential results (health-care provider discretion)**<br>Benin (18 years)[60], Botswana (no age limit)[40], Burkina Faso (18 years, but "mature minor" exception from 15 years)[61], Ethiopia (15 years, but "mature minor" exception from 13 years)[51], Ghana (16 years)[57], Liberia (14 years)[49], Malawi (13 years)[49], Mozambique (12 years)[42], Namibia (14 years)[50], Sierra Leone (18 years)[66], Somalia (15 years)[54], South Africa(12 years)[44], South Sudan (18 years)[67], Zimbabwe (16 years)[73]	13
**At risk of contracting HIV (e.g. sexually active)**<br>Burkina Faso (18 years, but "mature minor" exception from 15 years)[61], Liberia(14 years)[49], Namibia(14 years)[50], Sierra Leone(18 years)[66], Somalia (15 years)[54], Sudan (age of "minor" not defined)[39], United Republic of Tanzania (15 years)[71], Zimbabwe (16 years)[73]	9
**Symptomatic**<br>Benin (18 years)[60], Liberia (14 years)[49], Zimbabwe (16 years)[73]	3
**Pregnant**<br>Benin (18 years)[60], Botswana(no age limit)[40], Burkina Faso (18 years, "mature minor" exception from 15 years)[61], Cameroon (age of minor not defined)[32], Ghana (16 years)[57], Liberia (14 years)[49], Mozambique(12 years)[42], Namibia (14 years)[50], Rwanda (12 years)[43], Sierra Leone(18 years)[66], Somalia(15 years)[54], South Sudan (18 years)[67], United Republic of Tanzania (15 years)[71], Zambia(16 years)[58], Zimbabwe (16 years)[59]	15
**Parent (adolescent is already a parent)**<br>Benin (18 years)[60], Botswana (no age limit)[40], Cameroon (age of minor not defined)[32], Ghana (16 years)[57], Mozambique (12 years)[42], Namibia (14 years)[50], Sierra Leone (18 years)[66], Somalia (15 years)[54], South Sudan (18 years)[67], Zambia (16 years)[58]	10
**Head of household**<br>Central African Republic (18 years)[62], Liberia (14 years)[49], Mozambique (12 years)[42], Zambia (16 years)[58]	4
**Married**<br>Benin (18 years)[60], Botswana (no age limit)[40], Burkina Faso (18 years, "mature minor" exception from 15 years)[61], Ghana (16 years)[57], Liberia (14 years)[49], Mozambique (12 years)[42], Namibia (14 years) [33], Sierra Leone (18 years)[66], South Sudan (18 years)[67], United Republic of Tanzania (15 years)[55 71] Zambia (16 years)[58]	11
**Sex workers**<br>Liberia (14 years)[49], Rwanda (12 years)[43], Sierra Leone (18 years)[66], Somalia (15 years)[54], South Sudan (18 years)[67], Zambia (16 years)[58]	6
**Street children**<br>Liberia (14 years)[49]	1
**Emancipated minor**<br>Botswana (no age limit)[40], Central African Republic (18 years)[62], Kenya (15 years)[52], Liberia (14 years)[49], Madagascar (18 years)[64], South Sudan (18 years)[67]	6

For peer review only

SUPPLEMENTARY FILES

Included policies

Age of consent to HIV testing policies in 49 sub-Saharan African countries

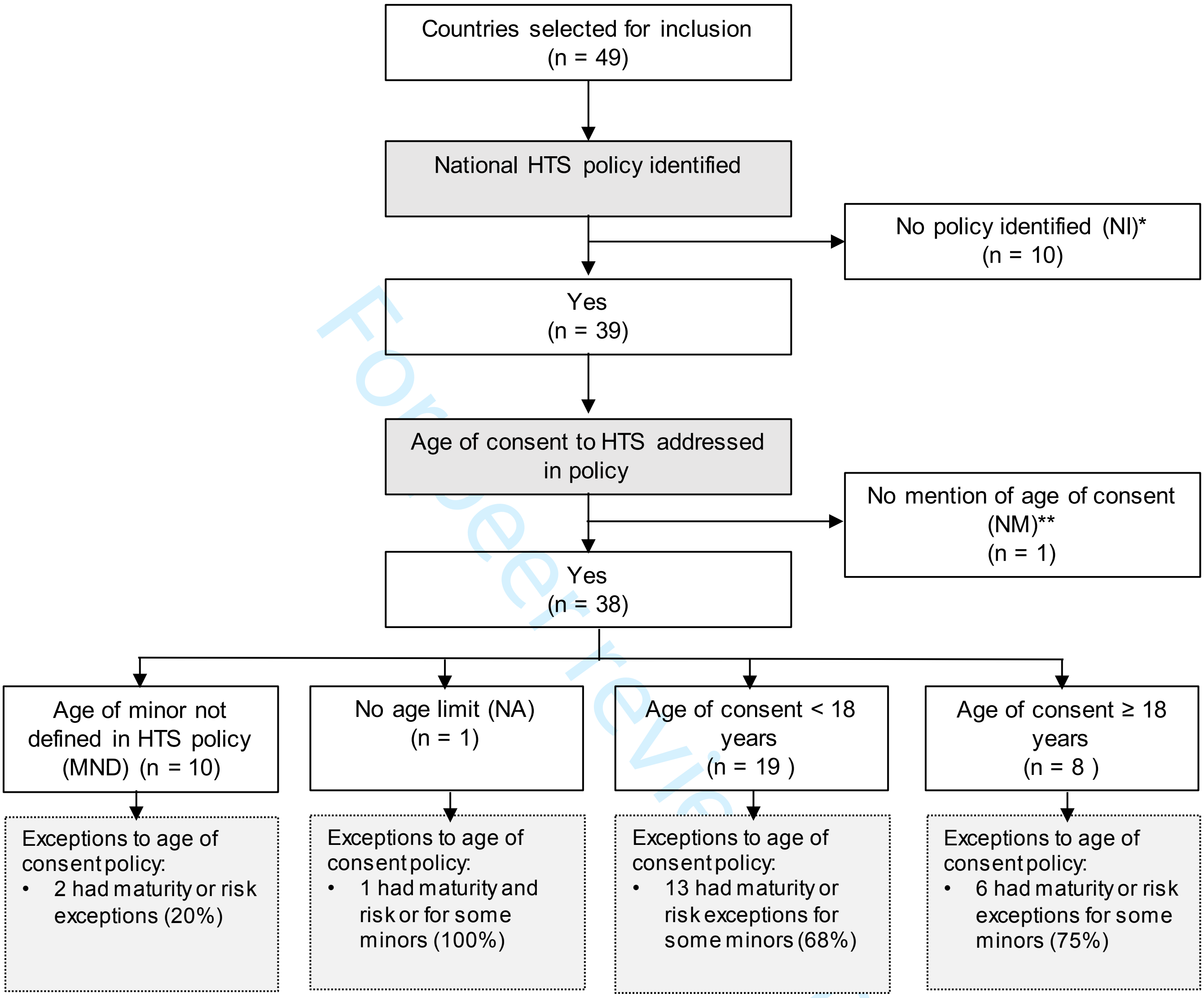

For peer review only

Search Strategy

The process for collecting HTS guidelines included the following methods:

1. Search of internal WHO database for HIV Testing Services (HTS) policy documents

2. Country by country search of AIDSFree HTS policy database (https://aidsfree.usaid.gov/resources/guidance-data/hts)

3. Country by country search of IAPAC/HIV Policy Watch website (http://www.hivpolicywatch.org/database.html)

4. Broad Google search for HTS policies from each WHO country using the following key words in English, French, Portuguese, and Spanish:
    - country name AND "HIV testing" AND policy
    - country name AND "HIV testing" AND guideline
    - country name AND PrEP AND policy
    - country name AND PrEP AND guideline
    - country name AND "pre-exposure prophylaxis" AND policy
    - country name AND "pre-exposure prophylaxis" AND guideline

Policy data extraction tool

Two reviewers extracted policy data using a standardised tool. The tool included the following:

1. Country name
2. WHO priority country (yes/no)
3. Sub-region (ESA, WCA)
4. HIV testing policy identified? /yes/no, for HIVST policies, document separately)
    - Type of HIV testing policy document (HTS, NSP, ART, PMTCT, other)
    - Document name, primary data source
    - Notes about document and or secondary data sources
    - HIV prevalence (≥5%, <5%)
    - Year of publication
    - Language
5. HTS policy classification
    - Law or policy existing for HTS, age of consent clearly defined, including no age limit (0/1)
    - Law or policy existing for HTS, but no age of consent specified (0/1)
    - Law or policy existing for HTS, but no mention of 'adolescent' or 'minor' (0/1)
    - No law or policy for HTS
    - Age of minor not defined
    - No age limit – all or most adolescents eligible
6. Age of consent for HIV testing (including policy page reference)
7. Exceptions
    - Shows maturity and understanding of the process and potential results, health-care provider discretion
    - At risk of contracting HIV
    - Symptomatic
    - Pregnant
    - Parent, adolescent is already a parent
    - Head of household
    - Married
    - Commercial sex worker
    - Street child
    - Emancipated minor
    - Notes concerning exceptions

(continued on following page)

For peer review only - http://bmjopen.bmj.com/site/about/guidelines.xhtml

Policy data extraction tool—continued

8. HIVST policy identified? (yes/no)
    - Type of HIVST policy doc (HTS, NSP, ART, other)
    - HIVST Document name (primary HIVST data source)
    - Notes about HIVST document and or secondary HIVST data source
    - Year of publication
    - Language
9. HIVST policy classification
    - HIVST law or policy existing, age of consent clearly defined (including no age limit)
    - HIVST law or policy existing, but no age of consent specified for HIVST
    - HIVST law or policy existing, but no mention of 'adolescent' or 'minor'
    - No law or policy for HIVST
    - No age limit – all or most adolescents eligible for HIVST
10. Age of consent for HIVST (including page reference)
11. Notes and exceptions for age of consent to HIVST
12. Age of consent as reported to NCPI (http://lawsandpolicies.unaids.org/)

For peer review only

BMJ Open

Adolescents and age of consent to HIV testing: an updated review of national policies in sub-Saharan Africa

Journal:	*BMJ Open*
Manuscript ID	bmjopen-2021-049673.R1
Article Type:	Original research
Date Submitted by the Author:	23-Jun-2021
Complete List of Authors:	Barr-DiChiara, Magdalena; World Health Organization, Global HIV, Hepatitis and STI Programmes<br>Tembo, Mandikudza ; London School of Hygiene and Tropical Medicine, Department of Infectious Disease Epidemiology; Biomedical Research and Training Institute<br>Harrison, Lisa; Public Health England, South West Screening and Immunisations<br>Quinn, Caitlin; World Health Organization, Global HIV, Hepatitis and STI Programmes<br>Ameyan, Wole ; World Health Organization, Global HIV, Hepatitis and STI Programmes<br>Sabin, Keith; UNAIDS<br>Jani, Bhavin; World Health Organization<br>Jamil, Muhammad; World Health Organization, Global HIV, Hepatitis and STI Programmes<br>Baggaley, R; World Health Organization, Global HIV, Hepatitis and STI Programmes<br>Johnson, Cheryl; World Health Organization, Global HIV, Hepatitis and STI Programmes
**Primary Subject Heading**:	HIV/AIDS
Secondary Subject Heading:	Health policy, Sexual health, Reproductive medicine, Public health, Paediatrics
Keywords:	International health services < HEALTH SERVICES ADMINISTRATION & MANAGEMENT, HIV & AIDS < INFECTIOUS DISEASES, PAEDIATRICS

SCHOLARONE™
Manuscripts

I, the Submitting Author has the right to grant and does grant on behalf of all authors of the Work (as defined in the below author licence), an exclusive licence and/or a non-exclusive licence for contributions from authors who are: i) UK Crown employees; ii) where BMJ has agreed a CC-BY licence shall apply, and/or iii) in accordance with the terms applicable for US Federal Government officers or employees acting as part of their official duties; on a worldwide, perpetual, irrevocable, royalty-free basis to BMJ Publishing Group Ltd ("BMJ") its licensees and where the relevant Journal is co-owned by BMJ to the co-owners of the Journal, to publish the Work in this journal and any other BMJ products and to exploit all rights, as set out in our licence.

The Submitting Author accepts and understands that any supply made under these terms is made by BMJ to the Submitting Author unless you are acting as an employee on behalf of your employer or a postgraduate student of an affiliated institution which is paying any applicable article publishing charge ("APC") for Open Access articles. Where the Submitting Author wishes to make the Work available on an Open Access basis (and intends to pay the relevant APC), the terms of reuse of such Open Access shall be governed by a Creative Commons licence – details of these licences and which Creative Commons licence will apply to this Work are set out in our licence referred to above.

Other than as permitted in any relevant BMJ Author's Self Archiving Policies, I confirm this Work has not been accepted for publication elsewhere, is not being considered for publication elsewhere and does not duplicate material already published. I confirm all authors consent to publication of this Work and authorise the granting of this licence.

For peer review only

Adolescents and age of consent to HIV testing: an updated review of national policies in sub-Saharan Africa

Magdalena Barr-DiChiara[1§], Mandikudza Tembo[2,3], Lisa Harrison[4], Caitlin Quinn[1], Wole Ameyan[1], Keith Sabin[5], Bhavin Jani[6], Muhammad S. Jamil[1], Rachel Baggaley[1], Cheryl Johnson[1]

1 Global HIV, Hepatitis and STI Programmes, World Health Organization, Geneva, Switzerland

2 MRC International Statistics and Epidemiology Group, London School of Hygiene and Tropical Medicine, London, United Kingdom

3 Biomedical Research and Training Institute, Harare, Zimbabwe

4 Public Health England, South West Screening and Immunisation Team, Bristol, United Kingdom

5 UNAIDS, Geneva, Switzerland

6 World Health Organization, Dar es Salaam, United Republic of Tanzania

§ Corresponding author:

Madgalena Barr-DiChiara
Department of Global HIV, Hepatitis, and STI Programmes
World Health Organization
Avenue Appia 20
1211 Geneva, Switzerland
+41 079 924 9388
barrdichiaram@who.int

E-mail addresses of authors:

MD: m.dichiara@gmail.com

MT: mandikudza.tembo@lshtm.ac.uk

LH: lisaharrisonwk@gmail.com

WA: ameyanw@who.int

RB: baggaleyr@who.int

CQ: quinnc@who.int

MJ: mjamil@who.int

CJ: johnsonc@who.int

Abstract

Objectives

In sub-Saharan Africa (SSA) where HIV burden is highest, access to testing, a key entry point for prevention and treatment remains low for adolescents (aged 10-19). Access may be hampered by policies requiring parental consent for adolescents to receive HIV testing services (HTS). In 2013, WHO recommended countries review HTS age of consent policies. Here, we investigate country progress and policies on age of consent for HIV testing.

Design

Comprehensive policy review.

Data sources

Policies addressing HTS were obtained through searching WHO repositories, governmental and non-governmental websites, and consulting country and regional experts.

Eligibility Criteria

HTS policies published by SSA governments before 2019 which included age of consent.

Data extraction and synthesis

Data were extracted on HTS age of consent including exceptions based on risk and maturity. Descriptive analyses of included policies were disaggregated by eastern and southern Africa (ESA) and western and central Africa (WCA) sub-regions.

Results

Thirty-nine policies were reviewed, 38 were eligible; 19/38 (50%) permitted HTS for adolescents ≤16 years without parental consent. Of these, six allowed HTS ≥12 years, two at ≥13, two at ≥ 14, five at ≥15, four at ≥ 16. In ESA, 71% (n=15/21) allowed those of ≤16 years to access HTS while only 24% (n=6/25) of WCA countries allowed the same. Maturity exceptions including marriage, sexual activity, pregnancy, or key population were identified in 18 policies. In 2019, 63% (n=19/30) of policies with clear age-based criteria allowed adolescents of 12-16 years to access HIV testing without parental consent, an increase from 37% (n=14/38) in 2013.

Conclusions

While many countries in SSA have revised their HTS policies, many do not specify age of consent. Revision of SSA consent to HTS policies, particularly in WCA, remains a priority to achieve the 2025 goal of 95% of people with HIV knowing their status.

STRENGTHS AND LIMITATIONS OF THIS STUDY

For peer review only - http://bmjopen.bmj.com/site/about/guidelines.xhtml

- This study provides a comprehensive review of national age of consent to HIV testing policies in sub-Saharan Africa.
- Despite a robust search approach, some policy documents may not have been identified.
- Due to varied document formats, the reviewers employed a non-automatized data extraction that was complemented by using key words to maintain repeatability.
- This review included national policy documents but did not assess national laws which contribute to a potentially limiting operational context to better understand guidance which informs routine implementation.

For peer review only

BACKGROUND

In the past decade, substantial but unequal progress has been made toward the global "90-90-90" fast-track goals: In 2019, an estimated 81% of people living with HIV (PLHIV) know their status, 82% of aware PLHIV are on antiretroviral therapy (ART) and 88% of PLHIV on ART are achieving viral suppression.[1] Despite successes, nearly 19% of the 38 million people living with HIV remain undiagnosed and there are an estimated 1.7 million new HIV infections annually.[2]

Globally, three-quarters (approximately 130,000) of the new 170,000 annual HIV infections among adolescents (aged 10-19 years) are in girls.[3] In sub-Saharan Africa (SSA), home to nearly 90% of all adolescents and young PLHIV, adolescent girls and young women (AGYW) aged 15-24 years make up one quarter (about 330,000) of the new HIV infections although they represent just 10% of the population.[2] The number of young people in SSA is rapidly increasing and it is estimated that there will be almost 500 million 15-24-year-olds in the region by 2055.[2] Recent projections point to declines in HIV infections among young people in SSA but not enough to reach global targets for low HIV incidence by 2030.[2 4]

Despite the high burden and incidence, and growing population, access to and uptake of HIV testing services (HTS), a pathway to essential prevention and treatment, remains remarkably low among adolescents.[5 6] In eastern and southern Africa (ESA) it is estimated only 27% of adolescent girls and 16% of adolescent boys aged 15-19 received an HIV test in 2019 and lower uptake was observed in western and central Africa(WCA)[3]. Access may be hampered by polices requiring parental consent for adolescents to receive HIV testing services (HTS).[7-9]

Adolescence is a time of rapid physical, sexual and cognitive development. Health services for adolescents, including HTS, should be delivered through youth-friendly approaches that reflect their diverse and evolving needs.[10] Social determinants, such as wealth and gender inequality as well as access to health and education services, impact adolescent health behaviour and outcomes in the near term, as they transition into adulthood and throughout the life course.[11-14]

Particularly underserved are adolescents and young people from key populations: men who have sex with men, transgender people, young people who sell sex[1] (people 10–24 years of age, including children 10–17 years who are sexually exploited and adults of 18–24 years who are sex workers), people who inject drugs, and people in prisons or closed settings. Key populations, especially those of younger ages, are criminalized and face stigma and discrimination from community members and providers.[15-17]

Adolescents at high ongoing risk who do not know their HIV status can benefit from HTS, prevention or treatment but access to these services is limited by policies requiring authorization from a parent or guardian.[7-9 18] While parental support can have a positive effect on adolescent's sexual health decision-making, for many adolescents who fear disclosing their sexual and other risk behaviours to their parents, those who do not have positive parental relationships, or for those who live in child-headed households, parental consent requirements pose a major barrier to HTS.[19] Empowering adolescents to freely access HTS and to choose how they want to test, including to use HIV self-testing (HIVST), supports equitable service delivery and the right to access health services.[20-22] Earlier analysis of the relationship between HTS coverage of young people in SSA and age of consent policies showed that allowing free adolescent access was associated with testing coverage of 14.0 percentage points higher for girls and 6.9 percentage points for boys.[8] In 2013, WHO guidance on HIV and adolescents encouraged countries to consider revising parental consent requirements to reduce barriers to HIV testing services.[23] Since that time, WHO

[1] While young adults who sell sex are considered sex workers, multiple international conventions describe the participation of those under 18 years of age in selling sex as a contravention of human-rights law, and those who do so are considered sexually exploited.

United Nations. United Nations Convention on the Rights of the Child (Article 1). U.N. Doc. A/Res/44/25, 1989 (http://www.ohchr.org/EN/ProfessionalInterest/Pages/CRC.aspx

United Nations. United Nations protocol to prevent, suppress, and punish trafficking in persons, especially women and children. Supplementing the United Nations convention against transnational organized crime. Article 3 (a-d); 2001 (A/45/49, http://www.unodc. org/documents/treaties/UNTOC/Publications/TOC%20Convention/TOCebook-e.pdf

For peer review only - http://bmjopen.bmj.com/site/about/guidelines.xhtml

has recommended HIVST, a convenient and private option which has been demonstrated highly acceptable for adolescents in SSA.[24 25] Countries in SSA, have moved to rapidly introduce and scale-up differentiated HIVST approaches that link people to diagnosis, treatment and care. Recent findings show that when given the option of facility-based HTS or HIVST, adolescents and young people choose HIVST over conventional modalities, and are more likely to test and share tests with peers and partners when using HIVST.[26 27]

Here we review policies in force following the 2013 WHO guidelines to evaluate country progress and uptake of WHO guidance on age of consent for HIV testing, including the age of consent for accessing HIVST.[7 23 28]

METHODS

Search strategy

We carried out a search of national policy documents related to HTS through searching WHO repositories, governmental and non-governmental websites and search engines, and contacting WHO country contacts and regional experts through April 2018 and updated our database through August 2020 (online supplementary file 1).[29]

Inclusion criteria

All national policy documents pertaining to HTS were included; national HIV guidelines and strategic plans covering HIV and integrated guidelines on the prevention, treatment and care of HIV. For inclusion in the review, the policy must have included adolescent consent to HIV testing and be published by a WHO member state in SSA, of which there were 49. The policy language was not restricted but in policies other than English, extraction of age of consent was prioritized. The date of publication was not restricted and when more than one policy was identified for a country, the most current publication was included. When necessary, we contacted government officials, regional technical advisors, and other key experts to gather and/or validate information.

Data extraction

For peer review only

Policy data were extracted using a standardized Excel based form (see supplementary file 2, policy data extraction tool). Information extracted included age of consent to HIV testing and/or self-testing as well as exceptions, including but not limited to individual maturity, key population status, emancipation, parenthood, marital status, sexual activity, and year of publication. HIV burden for included countries was classified as high (≥5%) or low (<5%) and collected from the Global AIDS Monitoring tool.[1 18] In addition to the policy assessment by the review team, national age of consent to HTS was also obtained from the National Commitments and Policy Instrument (NCPI) database, a global survey of HIV laws and policies.[28] Policies in languages other than English were reviewed with the aid of the translation software, Google Translate.

Two authors (MBD and MT) extracted information from policy documents independently.

Key word searching for terms including adolescent, child, minor, parent, and consent, was used to identify content addressing age of consent with full-text review completed on the sections identified through this process. Disagreements between reviewers were resolved by discussion and consultation with a senior author(CJ).

Analysis

Countries were classified according to two sub-regions: eastern and southern Africa (ESA) and western and central Africa (WCA)[2]. Policy data were then categorized using previously developed definitions and categories (Table 1): no mention, no adolescent access without parental consent, some adolescents may access without parental consent, and no minimum age limit.[8]

Table 1. Definitions for country policies to age of consent to HIV testing

[2] We present data in two sub regions, ESA and WCA, which closely align with UNAIDS regions. We include Djibouti and Somalia, which are not in these UNAIDS regions, We do not include Algeria, which is traditionally north Africa, though it sits in the WHO African region.

Policy category	Definition
No mention (NM)	Policy does not mention adolescents or minors or age of consent for HTS.
Minor not defined (MND)	Age of minor not defined.
Parental consent required (PCR)	Age of consent to HIV testing is 18 years or above. Ability to consent is defined by age.
Some adolescents may access without parental consent (SAC)	Age of consent to HIV testing is below 18 years. Minimum age to consent for HTS varies by country. Ability to consent is defined by age.
No minimum age limit (NA)	There is no minimum age limit for independent consent to HIV testing.
Mature minor exceptions (MME)	Minors classified as mature may access to HIV testing without parental consent. Criteria for exceptions to minimum age of consent may include:<br>• Shows maturity and understanding of the process and potential results (health-care provider discretion)<br>• At risk of contracting HIV or sexually active<br>• Symptomatic<br>• Pregnant<br>• Parent (adolescent is already a parent)<br>• Head of household<br>• Married<br>• Child engaged in commercial sex work<br>• Street child<br>Mature minor exceptions are not mutually exclusive of other approaches including: no adolescent access, some adolescent access, or no age limit.

We conducted a descriptive analysis, complementing 2019 policy data with results reported in NCPI. We assessed country policy changes since 2013, including variation in age of consent to HIV testing and maturity exceptions, by comparing our results to the previous WHO review on age of consent.[1 7]

Patient and public involvement

Patients and/or the public were not involved in the design, or conduct, or reporting of this research.

RESULTS

A total of 39 national policies published from 2005 through 2019 were identified among SSA countries, for ten countries no policy was identified. Of these, 1/39 (<3%) did not mention (NM) consent for adolescents or minors. 38 policies described consent for HTS and maturity exceptions and were included in the analysis: 10 policies did not provide definition of a minor (MND), one did not use age-based criteria for consent, and 27 policies detailed age-based of consent criteria (see supplementary file 3, included policies). Among included policies, eight limited HTS access without parental consent to persons ≥18 years

For peer review only - http://bmjopen.bmj.com/site/about/guidelines.xhtml

of age while 19 (50%) permitted HTS for adolescents < 18 years without parental consent. Of these, six allowed HTS ≥12 years, two at ≥13, two at ≥ 14, five at ≥15, four at ≥16 (Table 2).

The minimum age of consent was 12 years, reported in six countries, most of which were high burden (Lesotho, Mozambique, Rwanda, South Africa, Eswatini, and Uganda) (Table 2).

For peer review only

Table 2. Age of consent to HTS policies in sub-Saharan Africa

**National policies reviewed (n=49) [categories not mutually exclusive]**	**No. of countries (%)**
Policy existing for HTS, age of consent clearly defined (including no age limit)	30 (61.2%)
Policy existing for HTS, but no age of consent specified*	8 (16.3%)
Total number of countries with policies discussing age of consent to HTS	*38 (77.6%)*
Policy existing for HTS, but no mention of 'adolescent' or 'minor'	1 (2.0%)
No policy for HTS	10 (20.4%)
Total number of countries either without HTS policies, or that have policies that do not mention adolescents or minors	*11 (22.4%)*
**National age of consent to HTS (n=38)**<br>The year following the country indicates the year of publication for national policy.	**No. of countries (%)**
**Age of minor not defined**<br>Angola, 2015[30]; Burundi, 2014[31]; Cameroon, 2015[32]; Cape Verde, 2005[33]; Chad, 2011[34]; Comoros, 2007[35]; DRC, 2017[36]; Guinea, 2018[37]; Nigeria, 2016[38]; Sudan, 2016[39]	10 (26.3%)
**No age limit – all or most adolescents eligible****<br>Botswana, 2016[40]	1 (2.6%%)
**12 years**<br>Lesotho 2016[41]; Mozambique, 2015[42]; Rwanda, 2016[43]; South Africa, 2016[44]; Eswatini, 2018[45]; Uganda, 2016[46]	6 (15.8%%)
**13 years**<br>Gambia, 2014[47]; Malawi, 2016[48]	2 (5.3%%)
**14 years**<br>Liberia, 2015[49]; Namibia, 2016[50]	2 (5.3%)
**15 years**<br>Ethiopia, 2017[51]; Kenya 2015[52]; Senegal, 2017[53]; Somalia, 2017[54]; United Republic of Tanzania, 2019[55]	5 (13.2%)
**16 years**<br>Côte d'Ivoire, 2019[56]; Ghana, 2017[57]; Zambia, 2016[58]; Zimbabwe, 2014[59]	4 (10.5%)
**18 years**<br>Benin, 2017[60]; Burkina Faso, 2008[61]; Central African Republic, 2010, [62]; Eritrea, 2019[63]; Madagascar 2011[64]; Mali, 2017[65]; Sierra Leone, 2017[66]; South Sudan 2017[67]	8 (21.1%)

* These countries used the term 'minor' in their guidance, but do not specify an age.
** These countries stipulate considerations other than age to allow access to HTS (e.g. shows maturity, of reproductive age, or married, pregnant, or engaged in behaviour that would put them at risk).

Among high burden countries, 80% (n=8/10) allowed younger adolescents to access testing independent of parents/guardians (Botswana, NA; Eswatini,12; Lesotho, 12; Malawi, 13; Mozambique, 1; Namibia, 14; South Africa, 12; Uganda, 12) while 20% (n=2/10) limited independent consent to older adolescents (Zambia, 16; Zimbabwe 16), (Table 3).

Table 3. HIV testing policies identified, and age of consent to HTS in sub-Saharan Africa

	Country	Age of consent to HTS	Sub-region	HIV burden*
1	Angola	MND[30]	ESA	Low
2	Benin	18 (Age 15 if adolescent shows maturity)[60]	WCA	Low
3	Botswana	NA[40]	ESA	High
4	Burkina Faso	18 (MME: from 15 years of age.)[61]	WCA	Low
5	Burundi	MND[68]	WCA	Low
6	Cameroon	MND (MME: HTS from age 10-13 with parental consent, HTS from age 14-19 if sexually active or family head)[32]	WCA	Low
7	Cape Verde	MND [33]	WCA	Low
8	Central African Republic (the)	18 (MME: if there is no parent or guardian and a child is able to manage the decision, a minor may access HTS independently)[62]	WCA	Low
9	Chad	MND[34]	WCA	Low
10	Comoros (the)	MND[35]	ESA	Low
11	Congo (the)	NI	WCA	Low
12	Côte d'Ivoire	16[69]	WCA	Low
13	Democratic Republic of the Congo	MND[36]	WCA	Low
14	Djibouti	NI	ESA	Low
15	Equatorial Guinea	NI	WCA	High
16	Eritrea	18[63]	ESA	Low
17	Eswatini	12 (MME: people of 16 years or more may access HIVST)[45]	ESA	High
18	Ethiopia	15 (MME: mature minors of 13-15 years may consent to HIV testing, those less than 15 years of age cannot consent to HIV testing) [51]	ESA	Low
19	Gabon	NI	WCA	Low
20	Gambia (the)	13 (MME: for those below age 13, parental consent is required unless it is an emergency)[47]	WCA	Low
21	Ghana	16 (MME: any person who is between 14 and 16 years and is sexually active, married, pregnant, a parent or requests HTS is considered to be a mature minor and is able to give full informed consent)[57]	WCA	Low
22	Guinea	MND[37]	WCA	Low
23	Guinea-Bissau	NI	WCA	Low
24	Kenya	15 (MME: emancipated minor exception, survivors of sexual violence who are under the age of 15 may access HIV testing without parental consent)[52]	ESA	Low
25	Lesotho	12[41]	ESA	High
26	Liberia	14 (MME: any child who is married, pregnant, works as a commercial sex worker, is a street teenager, family head or with a history of sexual intercourse is regarded as a mature or emancipated minor and can consent for HIV testing)[49]	WCA	Low
27	Madagascar	18[64]	ESA	Low

For peer review only

	Country	Age of consent to HTS	Sub-region	HIV burden*
28	Malawi	13 (MME: minors of fewer than 13 years of age may consent to HTS)[48]	ESA	High
29	Mali	18[65]	WCA	Low
30	Mauritania	NI	WCA	Low
31	Mauritius	NM[70]	ESA	Low
32	Mozambique	12 (MME: adolescents married, living as if married, sexually active, head of household may give consent for HIV testing)[42]	ESA	High
33	Namibia	14[50]	ESA	High
34	Niger (the)	NI	WCA	Low
35	Nigeria	MND [38]	WCA	Low
36	Rwanda	12[43]	ESA	Low
37	São Tomé and the Príncipe	NI	WCA	Low
38	Senegal	15[53]	WCA	Low
39	Seychelles	NI	ESA	Low
40	Sierra Leone	18 (MME: child survivors of sexual assault or abuse, married young people under age 18, pregnant, parents, minors engaged in behaviour which puts them at risk, child sex workers)[66]	WCA	Low
41	Somalia	15 (MME)[54]	ESA	Low
42	South Africa	12 (For HIVST, children between age 12 and 18 years should use directly assisted testing options)[44]	ESA	High
43	South Sudan	18 (MME: married, pregnant, parents, emancipated)[67]	ESA	Low
44	Sudan	MND (MME: adolescent KP)[39]	ESA	Low
45	Togo	NI	WCA	Low
46	Uganda	12[46]	ESA	High
47	United Republic of Tanzania (the)	15[55 71]	ESA	Low
48	Zambia	16[58]	ESA	High
49	Zimbabwe	16 (MME: shows maturity, at risk of HIV, symptomatic, pregnant)[59]	ESA	High

MND: Minor not defined
NA: No age of consent to HTS described in policy
NI No information
NL: No age of consent to sex law

*Countries were classified as low- and high- HIV burden using an HIV prevalence threshold of 5%. Data was obtained from Global AIDS Monitoring, 2020 [1].

All high burden country policies reviewed permitted adolescents of 16 and younger to access HTS without parental consent pointing to alignment with WHO 2013 guidance and movement toward accessible testing services for adolescents where HIV is a major public health threat. NCPI age of consent policy reporting complemented our findings; however, some instances of disagreement were identified (Table 4).

For peer review only

Table 4. Age of consent to HTS in sub-Saharan Africa national policies, 2013, 2019, and the National Commitments and Policy Instrument (NCPI)

Age of consent policy	WHO 2013* (n = 33)		WHO 2019** (n = 38)		NCPI*** (n= 49)	
	Name of countries	No. of countries (%)	Name of countries	No. of countries (%)	Name of countries	No. of countries (%)
No information/No policy identified, (NCPI: no data)	Chad, The Comoros, Equatorial Guinea, Eritrea, Gabon, The Gambia, São Tomé and the Príncipe, South Sudan	8	Congo, Djibouti, Equatorial Guinea, Gabon, Guinea-Bissau, Mauritania, Niger, Sao Tome and Principe, Seychelles, Togo	10	Cabo Verde, Cameroon, Chad, Comoros, Eritrea Guinea-Bissau, Sao Tome and Principe, Somalia	7 (14.2%)
Policy existing for HTS, but no mention of 'adolescent' or 'minor'	Mauritania	1 (2.1%)	Mauritius[70]	1 (2.6%)	-	-
Age of minor not defined	Angola, Benin, Burundi, Guinea-Bissau, Madagascar, Mali, Togo	7 (17.5%)	Angola[30], Burundi[31], Cameroon[32]¸ Cape Verde, Chad[34], Comoros[35], DRC[36], Guinea[37], Nigeria[38], Sudan[39]	10 (26.3%)	-	-
No age limit – all or most adolescents eligible	Botswana, Cape Verde, Kenya, Mauritius, Somalia (Somaliland)	5 (12.5%)	Botswana[40]	1 (2.6%)	-	-
12 years (NCPI: yes, for adolescents younger than 12 years)	Lesotho, South Africa, Uganda	3 (7.5%)	Lesotho[72], Mozambique[42], Rwanda[43], South Africa[44], Eswatini[45], Uganda[46]	6 (15.8%)	Rwanda	1 (2.0%)
13 years	Malawi	1 (2.5%)	Gambia[47], Malawi[48]	2 (5.3%)	-	0
14 years (NCPI: yes, for adolescents younger than 14 years)	Liberia	1 (2.5%)	Liberia[49], Namibia[50]	2 (5.3%)	Benin, Central African Republic, Congo, Gabon, Gambia, Lesotho, Liberia, Malawi, Mozambique, Namibia, Senegal, South Africa, Togo, Uganda	15 30.6%)
15 years	Ethiopia, Rwanda, Senegal	3 (7.5%)	Ethiopia[51], Kenya[52], Senegal[53], Somalia[54], United Republic of Tanzania[55]	5 (13.2%)	-	0
16 years (NCPI: yes, for adolescents younger than 16 years)	Congo, Mozambique, Namibia, Swaziland, Zambia, Zimbabwe	6 (15.0%)	Côte d'Ivoire[69], Ghana[57], Zambia[58], Zimbabwe[59]	4 (10.5%)	Botswana, Burundi, Cote d'Ivoire, Djibouti, Equatorial Guinea, Guinea, Niger, Zambia, Zimbabwe	9 (18.3%)

For peer review only

Age of consent policy	WHO 2013* (n = 33)		WHO 2019** (n = 38)		NCPI*** (n= 49)	
18 years<br>(NCPI: yes for adolescents less than 18 years)	Burkina Faso, Cameroon, Central African Republic, Côte d'Ivoire, Democratic Republic of the Congo, Djibouti, Ghana, Guinea, Niger, Nigeria, Seychelles, Sierra Leone, Sudan, United Republic of Tanzania	14 (35.0%)	Benin[60], Burkina Faso[61], Central African Republic[62], Eritrea[63], Madagascar[64], Mali[65], Sierra Leone[66], South Sudan[67]	8 (21.1%)	Angola, Burkina Faso, Democratic Republic of Congo, Eswatini, Ethiopia, Ghana, Kenya, Madagascar, Mali, Mauritius, Nigeria, Seychelles, Sierra Leone, South Sudan, Sudan, United Republic of Tanzania	17(35.6%)

*2013 review included both policies and laws while this review included only policies [7]

**Findings identified in the context of this policy review

***Data reported in the UNAIDS National Commitments and Policy Instrument, 2017, 2018, 2019

For peer review only

Maturity exceptions

Maturity exceptions that waive parental consent requirements and effectively lower the age of consent for some groups of minors were identified in more than half (n=20/38) of policies that address age of consent, including those policies that did not specify age-based definitions of the age of majority (Table 5).

Table 5. Exceptions for HTS below age of consent

**Exceptions for testing below stated legal age of consent** The age in parentheses indicates the national age of consent to HTS before the exception is applied.	**No. of countries**
**Shows maturity and understanding of the process and potential results (health-care provider discretion)** Benin (18 years)[60], Botswana (no age limit)[40], Burkina Faso (18 years, but "mature minor" exception from 15 years)[61], Ethiopia (15 years, but "mature minor" exception from 13 years)[51], Ghana (16 years)[57], Liberia (14 years)[49], Malawi (13 years)[49], Mozambique (12 years)[42], Namibia (14 years)[50], Sierra Leone (18 years)[66], Somalia (15 years)[54], South Africa(12 years)[44], South Sudan (18 years)[67], Zimbabwe (16 years)[73]	13
**At risk of contracting HIV (e.g. sexually active)** Burkina Faso (18 years, but "mature minor" exception from 15 years)[61], Liberia(14 years)[49], Namibia(14 years)[50], Sierra Leone(18 years)[66], Somalia (15 years)[54], Sudan (age of "minor" not defined)[39], United Republic of Tanzania (15 years)[71], Zimbabwe (16 years)[73]	9
**Symptomatic** Benin (18 years)[60], Liberia (14 years)[49], Zimbabwe (16 years)[73]	3
**Pregnant** Benin (18 years)[60], Botswana(no age limit)[40], Burkina Faso (18 years, "mature minor" exception from 15 years)[61], Cameroon (age of minor not defined)[32], Ghana (16 years)[57], Liberia (14 years)[49], Mozambique(12 years)[42], Namibia (14 years)[50], Rwanda (12 years)[43], Sierra Leone(18 years)[66], Somalia(15 years)[54], South Sudan (18 years)[67], United Republic of Tanzania (15 years)[71], Zambia(16 years)[58], Zimbabwe (16 years)[59]	15
**Parent (adolescent is already a parent)** Benin (18 years)[60], Botswana (no age limit)[40], Cameroon (age of minor not defined)[32], Ghana (16 years)[57], Mozambique (12 years)[42], Namibia (14 years)[50], Sierra Leone (18 years)[66], Somalia (15 years)[54], South Sudan (18 years)[67], Zambia (16 years)[58]	10
**Head of household** Central African Republic (18 years)[62], Liberia (14 years)[49], Mozambique (12 years)[42], Zambia (16 years)[58]	4
**Married** Benin (18 years)[60], Botswana (no age limit)[40], Burkina Faso (18 years, "mature minor" exception from 15 years)[61], Ghana (16 years)[57], Liberia (14 years)[49], Mozambique (12 years)[42], Namibia (14 years) [33], Sierra Leone (18 years)[66], South Sudan (18 years)[67], United Republic of Tanzania (15 years)[55 71] Zambia (16 years)[58]	11
**Sex workers** Liberia (14 years)[49], Rwanda (12 years)[43], Sierra Leone (18 years)[66], Somalia (15 years)[54], South Sudan (18 years)[67], Zambia (16 years)[58]	6
**Street children** Liberia (14 years)[49]	1
**Emancipated minor** Botswana (no age limit)[40], Central African Republic (18 years)[62], Kenya (15 years)[52], Liberia (14 years)[49], Madagascar (18 years)[64], South Sudan (18 years)[67]	6

For peer review only

The most common maturity exceptions were: pregnancy (n=15/38), demonstrated maturity and understanding of test (n= 13/38), marriage (n=11/38), parenthood (n=10/38), and being at risk of contracting HIV due to behaviours or population group (n=9/38). Only six countries (Liberia, Rwanda, Sierra Leone, Somalia, South Sudan, Zambia) include maturity exceptions for adolescents from key populations group, all related to people who sell sex. Only one country (Liberia) included a maturity exception for street children. One country (Botswana) does not use age to determine adolescent access to HIV testing. Instead, the policy emphasised the need for providers to assess client maturity and client risks.

When stratified by sub-region, we identified notable differences the age of consent policies between ESA (n=21 countries) and WCA (n=25 countries) subregions. In WCA, for 32% of countries (n=8/25), no HTS policy was identified (Congo, Equatorial Guinea, Gabon, Guinea-Bissau, Mauritania, Niger, São Tomé and the Príncipe, Seychelles, Togo). Whereas, in ESA only two countries (Seychelles and Djibouti) had no identified policy.

Of the 17 WCA countries for which a policy was identified, 70% (n=12/17) included clearly defined age of consent for HTS and 5 countries (Benin, Burkina Faso, Central African Republic, Mali, and Sierra Leone) required parental consent for adolescents under 18 of age to test. In ESA there were six countries with an age of consent of 12 years whereas none were identified in WCA.

Changes in age of consent policies for HIV testing over time

Thirty-one of the country policies included (Table 2) were published after the 2013 release of WHO guidance on HIV and adolescents which called on policy-makers to detail clear and specific age-based consent language permitting adolescent access to HTS.[23] In 2013, clear age-based criteria for consent to HTS were present in 70% (n=28/40) of the laws and polices reviewed, we identified a similar proportion of policies with clear age-based consent language in 2019, 73% (n=28/38).[7] We observed an increase in the proportion of polices with age-based consent criteria which permitted adolescents of 16 years or younger to access HTS without parental consent: in 2013 50% (n=14/28) allowed access without parental consent to those of 16 years or younger, and in 2019 68% (n=19/28) allowed access without parental consent to those of 16 years or younger. While a majority of policies had been updated since 2013, reduction in age of consent to HTS occurred in just six policies; in three high burden countries (Eswatini, Mozambique and Namibia) and just four low burden countries (Cote d'Ivoire, Gambia, Rwanda, United Republic of Tanzania).

For peer review only

HIV self-testing

No policies in the 2013 review included information on age of consent for HIVST. The first WHO recommendation on HIVST was introduced in 2016. In 2019, nearly half of (44%, 17/38) national policies included self-testing and 53% of these (n=9/17) clearly defined age of consent for access to HIVST. Among HVST policies, nine clearly defined age of consent for HIVST (Cote d'Ivoire, 16; Eritrea, 18; Eswatini, 16; Ghana, 16; Mali, 18; Somalia; 18, South Africa, 12 with directly assisted HIVST; South Sudan, 18, Tanzania, 18). Nine polices (Benin, Cameroon, Democratic Republic of Congo, Kenya, Lesotho, Malawi, Nigeria, Zambia and Zimbabwe) addressed HIVST but did not include clear definition of age of consent and among these were two standalone HIVST policies (Zambia and Zimbabwe). HIVST policies were diverse in format, some were standalone while others integrated HIVST into broader testing service polices. Two policies (Kenya and South Africa) explicitly enabled self-testing, but only with direct assistance for those under the age of 18 years, thus distinguishing HIVST from conventional provider delivered options. Eswatini and Tanzania and Somalia had a higher age of consent for HIVST (16 years for Eswatini, 18 years for Tanzania) than for provider delivered methods (12 years for Eswatini, 15 years for Tanzania and Somalia).

NCPI

NCPI, a mechanism for monitoring national laws and policies, captures age of consent regulations for HIV testing and other reproductive health services among other themes.[28 74] Our review findings complement the policy reporting in NCPI but do not capture consistent country-level information on age of consent to HIV testing (Table 3). The discrepancy of our findings with those reported by NCPI highlight challenges in aggregating policy information in surveys and research. Since NCPI includes age of consent policy information covering HTS, ART and sexual and reproductive health and rights (SRHR) services it provides regular regional intelligence and offers a platform to routinely review, validate and engage around age of consent policies.

DISCUSSION

Our review found that half of policies in SSA allowed adolescents aged 16 and younger to provide independent consent for HIV testing.

For peer review only

We found the minimum specified age of consent for HTS in any SSA country was 12 years with one country reporting no age- based criteria. In comparison, the minimum specified age of consent for HTS in western Europe and north America, as 14 years, with some countries reporting no age based criteria, and many countries not reporting.[28] There has been substantial progress in SSA since 2013 when WHO encouraged countries to review and adapt consent polices to make HIV testing accessible to adolescents, with 31 updated policies identified. Among these, seven lowered the age of consent.[23] Policy makers in SSA have moved to address HIVST but there is more work to be done in both developing policy and including clearly defined age of consent language. Our review also identified gaps in age of consent policies in SSA, particularly WCA: overall about a quarter of policies (n=10/38) did not clearly describe age of consent and 21% restricted HTS to those 18 years or above.

In this review we assessed only HIV testing policies. However, polices should facilitate integration and linkage to promote comprehensive adolescent health packages that deliver the benefits of HIV prevention, treatment, and other SRHR services so that public health impact can be realized. In countries with clear age of consent policies (including many in ESA), alignment of HTS policies with other SRHR and HIV prevention policies should be prioritized. In countries without polices, or in those with vague policies (including many in WCA) development of guidance with clear consent language that makes HIV testing, SRHR and HIV services broadly available to adolescents is needed. In all settings, key population and sexually active adolescents need policies that allow them to provide their own consent for HTS.[28] One recent country example of policy change took place in Tanzania, where in 2019 following a period of coordinated political advocacy, parliamentary support for lowering age of consent for HIV testing from 18 to 15 years was obtained; age of consent for HIV self-testing remained at 18 years of age.

A highlight of this review is the assessment of policies around HIVST, a highly acceptable way to engage adolescents in HIV testing and to make testing accessible for adolescents and young people.[75] In 2013, only two countries (Kenya and Unites States of America) had HIVST policies, and there were no WHO recommendations regarding their use. Further, the United States Food and Drug Administration restricted distribution by requiring proof of age for those accessing HIVST and limiting use to those of 17 years and older although initial studies included those of 14 years and older in sub-group analysis.[76]

For peer review only

Following the release of the first WHO guidance on self-testing for HIV in 2016, our 2019 review identified 17 country policies that addressed HIVST. Adolescents who self-test need tools and resources to support linkage to prevention and treatment services and approaches that deliver self-testing to this population through assisted and unassisted methods have been demonstrated effective and acceptable in SSA.[75] Two countries (South Africa and Kenya) stipulated assisted HIVST approaches for younger adolescents, thus making the modality available while ensuring a minimum standard of support for performing the self-test, interpreting the result, and linking to other services.

Policies frequently included maturity exceptions that eliminate parental consent requirements for adolescents seeking HTS, including those from key populations who are at particularly high risk of HIV and may also face social and structural barriers to testing, prevention and care services.[77]
Many other vulnerable adolescents are left out and overlooked: only six countries (Liberia, Rwanda, Sierra Leone, Somalia, South Sudan, Zambia) have policies that address adolescents who sell sex and exceptions for adolescents from other key populations were not identified. Exceptions did frequently address adolescent girls; those for marriage, pregnancy, involvement in sex work, and sexual activity may effectively lower age of consent for some girls, however, girls may not feel empowered to seek health services or to take a test when services are not designed to be accessible to adolescents. Overall, we found great variation in maturity exceptions and are not able to ascertain how these are interpreted and put into practices by implementers and service providers or how they ultimately impact access to HIV testing for adolescents. In settings where maturity exception policies are present, programmes should assess fidelity as well as seek and consider adolescent perspectives while they aim to strengthen service delivery for HIV testing and prevention.

Strengths and limitations

Documents from nearly forty countries were reviewed using a standard approach to extraction and analysis. Despite a robust approach, some policy documents may not have been identified. Due to varied document formats, the reviewers employed a non-automatized data extraction that was complemented by using key words to maintain repeatability. This review included national policy documents but did not assess national laws which contribute to a potentially limiting operational context to better understand guidance which informs routine implementation.[9] Since the review did not assess implementation, it does

For peer review only

not provide insight into how policies ultimately impact HIV testing service delivery and access for adolescents.

Further policy reviews and national assessments that address alignment on age of consent within and between HIV testing, prevention and treatment, STI and other sexual and reproductive health services will illuminate opportunities to strengthen policy and implementation. Finally, while a significant proportion of policies reviewed did not define the age of consent which limited assessment of regional trends, this did not hinder our ability to assess whether national policies contained clear description of age of consent to test.

CONCLUSIONS

Since 2013, 31 SSA countries have updated policies that include age of consent to HTS, with seven reducing the age of consent to 12 years or eliminating age-based criteria. Our findings suggest that adolescents in 19/38 (50%) of SSA countries may access HTS without consent of a parent or guardian at varying ages: 16, 15, 14, or 12 years. Access to HTS for adolescents remains limited in WCA, where policies are fewer, older, and have vague or no age of consent language. Adolescence, a period of rapid physical, sexual and cognitive development, is a time when young people need health services that address their diverse and evolving needs. HTS polices should acknowledge the right of adolescents to provide their own consent to testing, particularly through newly introduced approaches like HIVST, and be aligned with national SRHR guidance so that adolescents can receive the benefits of services for contraception, voluntary medical male circumcision, sexually transmitted infections, HIV prevention and antiretroviral treatment. Where adolescents have supportive parents or trusted older relatives, involving these in testing is often of great benefit to coping with a result and discussing linkage to other services post testing. As countries renew efforts to ensure that 95% of people with HIV know their status, and to improve testing, prevention and treatment access for adolescents, lowering or eliminating age of consent for HIV testing should continue to be prioritized.

For peer review only

ORCiD: Magdalena Barr-DiChiara https://orcid.org/0000-0002-7404-7843

List of abbreviations

AGYW: adolescent girls and young women

ART: antiretroviral therapy

ESA: eastern and southern Africa

HIVST: HIV self-testing

HTS: HIV testing services

MME: mature minor exceptions

MND: minor not defined

NA: no minimum age limit

NCPI: National Commitments and Policy Instrument

NM: no mention

PCR: parental consent required

PLHIV: people living with HIV

SAC: some adolescents may access without parental consent

SRHR: sexual and reproductive health and rights

DECLARATIONS

Ethics approval Not applicable.

Availability of data and materials Data may be obtained from a third party and some data are publicly available. Most policies included in this review are available through the following websites: (1) https://aidsfree.usaid.gov/resources/guidancedata/hts. (2) http://www.hivpolicywatch.org/database.html. If information on a policy cannot be found through these sources, please contact the authors of this review for additional information.

Competing interests The authors declare no competing interests. The contents in this article are those of the authors and do not necessarily reflect the view of the World Health Organization.

For peer review only

Funding Funding for this review was provided by the U.S. President's Emergency Plan for AIDS Relief (PEPFAR) through World Health Organization and USAID's Consolidated WHO Grant, Agreements US-2015-0839 and US-2016-940.

Patient consent for publication Not required.

Not commissioned; externally peer reviewed.

Contributors CJ and RB devised the review. MBD and CJ designed the protocol. MBD conducted the search, and MT conducted the screening and data extraction. CJ and RB supervised the review.

MBD and MT qualitatively synthesized the findings and conducted descriptive analyses. MBD and LH drafted the manuscript with input from MT, CJ and MJ. MBD, MT, LH, BJ, CQ, WA, MJ, KS, RB, CJ reviewed the draft, provided critical review, and read and approved the final manuscript.

Acknowledgements The authors thank all country and regional contacts for policies for the review.

For peer review only

REFERENCES

1. UNAIDS. Global factsheet 2019. Geneva: United Nations Joint Programme on HIV/AIDS, 2020.
2. UNAIDS. Siezing the moment, tackling entrenched inequalities to end epidemics: global AIDS update 2020. Geneva: United Nations Join Programme on HIV/AIDS, 2020.
3. UNICEF. UNICEF Data: Adolescent HIV prevention https://data.unicef.org/topic/hivaids/adolescents-young-people/: UNICEF; 2020 [Available from: https://data.unicef.org/topic/hivaids/adolescents-young-people/.
4. UNAIDS. Progress towards the start free, stay free, AIDS free targets. Geneva: United Nations Joint Programme on HIV/AIDS, 2020.
5. Wong V, Murray K, Phelps B, et al. Adolescents, young people, and the 90-90-90 goals: a call to improve HIV testing and linkage to treatment. *AIDS* 2017;31(Supplement 3):S191–S94. doi: https://doi.org/10.1097/QAD.0000000000001539
6. Kurth A, Lally M, Choko A, et al. HIV testing and linkage to services for youth. *Journal of the International AIDS Society* 2015;18(Suppl 1):19433. doi: https://doi.org/10.7448/IAS.18.2.19433
7. WHO. HIV and adolescents: guidance for HIV testing and counselling and care for adolescents living with HIV: annex 15: Adolescent consent to testing: a review of current policies and issues in sub-Saharan Africa. Geneva: World Health Organization, 2013.
8. McKinnon B, Vandermorris A. National age-of-consent laws and adolescent HIV testing in sub-Saharan Africa: a propensity-score matched study. *Bulletin of the World Health Organization* 2019;97(1):42–50. doi: https://doi.org/10.2471/BLT.18.212993
9. Eba P, Lim H. Reviewing independent access to HIV testing, counselling and treatment for adolescents in HIV-specific laws in sub-Saharan Africa: implications for the HIV response. *Journal of the International AIDS Society* 2017;20(1): 21456. doi: https://doi.org/10.7448/IAS.20.1.21456
10. WHO. WHO recommendations on adolescent sexual and reproductive health and rights. Geneva: World Health Organization, 2018.
11. Viner R, Ozer E, Denny S, et al. Adolescence and the social determinants of health. *Lancet* 2012;379(9826):1641-52. doi: doi: 10.1016/S0140-6736(12)60149-4
12. Stoner M, Nguyen N, Kilburn K, et al. Age-disparate partnerships and incident HIV infection in adolescent girls and young women in rural South Africa. *AIDS* 2019;33(1):83–91. doi: https://doi.org/10.1097/QAD.0000000000002037
13. Brown K, Williams DB, Kinchen S, et al. Status of HIV Epidemic Control Among Adolescent Girls and Young Women Aged 15–24 Years — Seven African Countries, 2015–2017. 2018; 67(1):29–32.
14. Starrs A, Ezeh A, Barker G, et al. Accelerate progress—sexual and reproductive health and rights for all: report of the Guttmacher–Lancet Commission. *Lancet* 2018;391 doi: http://dx.doi.org/10.1016/
15. Baggaley R, Armstrong A, Dodd Z, et al. Young key populations and HIV: a special emphasis and consideration in the new WHO Consolidated Guidelines on HIV Prevention, Diagnosis, Treatment and Care for Key Populations. *J Int AIDS Soc* 2015;18(2 S1)
16. Cornell M, Dovel K. Reaching key adolescent populations. *Current opinion in HIV and AIDS* 2018;13(3):274–80. doi: https://doi.org/10.1097/COH.0000000000000457
17. WHO. HIV and young people who sell sex. Geneva: World Health Organization 2015.
18. WHO. Consolidated guidelines on HIV testing services, 2019 Geneva: World Health Organization, 2019.
19. Sam-Agudu N, Folayan M, Ezeanolue E. Seeking wider access to HIV testing for adolescents in sub-Saharan Africa. *Pediatr Res* 2016;79(6):838–45. doi: doi: 10.1038/pr.2016.28
20. WHO, Institute RT. The voices, values and preference of adolescents on HIV testing and counselling: consultation for the development of the World Health Organization HIV testing and counselling guidelines for adolescents. Geneva: World Health Organization, Royal Tropical Institute, 2013.
21. Convention on the Rights of the Child. https://www.ohchr.org/en/professionalinterest/pages/crc.aspx, 1989.
22. General comment No. 4: Adolescent Health and Development in the Context of the Convention on the Rights of the Child: UN Committee on the Rights of the Child (CRC), 2003.
23. WHO. HIV and adolescents: guidance for HIV testing and counselling and care for adolescents living with HIV. Geneva: World Health Organization, 2013.
24. WHO. Guideline on HIV self-testing and partner notification: Supplement to consolidated guidelines on HIV testing services. Geneva: World Health Organization, 2016.
25. Hatzold K, Gudukeya S, Mutseta M, et al. HIV self-testing: breaking the barriers to uptake of testing among men and adolescents in sub-Saharan Africa, experiences from STAR demonstration projects in Malawi, Zambia and Zimbabwe. *Journal of the International AIDS Society* 2019;22(S1) doi: https://doi.org/10.1002/jia2.25244

For peer review only

26. Pettifor A, Lippman S, Kimaru L, et al. HIV self-testing among young women in rural South Africa: A randomized controlled trial comparing clinic-based HIV testing to the choice of either clinic testing or HIV self-testing with secondary distribution to peers and partners. *EClinical Medicine* 2020 doi: https://doi.org/10.1016/j.eclinm.2020.100327
27. Indravudh P, Sibanda E, d'Elbée M. 'I will choose when to test, where I want to test': investigating young people's preferences for HIV self-testing in Malawi and Zimbabwe. *AIDS* 2017;31(Suppl 3):S2013-S212. doi: doi: 10.1097/QAD.0000000000001516.
28. UNAIDS. UNAIDS Laws and Policies Analytics. http://lawsandpolicies.unaids.org/about: United Nations Joint Programme on HIV/AIDS, 2020.
29. Fonner V, Sands A, Figueroa C, et al. Country adherence to WHO recommendations to improve the quality of HIV diagnosis: a global policy review. *BMJ Global Health* 2020;5:e001939. doi: http://dx.doi.org/10.1136/bmjgh-2019-001939
30. Normas de Tratamento Antirretroviral. República De Angola: Ministerio da Saude, 2015.
31. Directives Nationales de Traitment Anti Retrovirale de L'Infection par au Burundi: Ministre de la Sante Publique et de la Lutte Contre le SIDA 2014.
32. National Guideline on the Prevention and Management of HIV in Cameroon. Government of the Republic of Cameroon: Ministry of Public Health, 2015.
33. Procedimentos, normas e direcrizes do atendimento das pessoas Cabo Verde: Direcao-Geral de Saude, Programa de Luta Contra a SIDA/IST, Tuberculose e Lepra, 2005.
34. Manuel de Formation en Counseling VIH/SIDA/IST. Republic du Tchad: Ministere de la Sante Publique, 2011.
35. Guide de prise en charge de l'infection à VIH Aux Comores. Union des Comores: Vice Présidence en charge du Ministère de la Santé , de la Solidarité 2007.
36. Guide de Prise en Charge Integree du VIH en Republique Democratique du Congo. Gouvernement de la République démocratique du Congo: Ministere de la Sante Publique, 2017.
37. Cadre strategique national de lutte contre le SIDA 2018-2022. Conakry, Guinea Comite National de Lutte Contre le SIDA, Secretariat Executif, , 2018.
38. National Guidelines for HIV Prevention Treatment and Care. Government of Nigeria: Federal Ministry of Health, 2016.
39. National Consolidated HIV Testing Services Guidelines. Government of Sudan: Federal Ministry of Health, 2016.
40. Handbook of the Botswana 2016 Integrated HIV Clinical Care Guidelines. Government of the Republic of Botswana: Ministry of Health, 2016.
41. National Guidelines on the Use of Antiretroviral Therapy for HIV Prevention and Treatment. Government of Lesotho: Ministry of Health, 2016.
42. Directriz nacional para a implementação do aconselhamento e testagem em saúde: República de Moçambique, Ministério da Saúde, Direcção Nacional de Assistência Médica, PNC ITS/HIV/SIDA, 2015.
43. National Guidelines for Prevention and Management of HIV and STIs. Government of the Republic of Rwanda: Ministry of Health, 2016.
44. National HIV Testing services: Policy. Government of the Republic of South Africa: Department of Health, 2016.
45. Swaziland Integrated HIV Management Guidelines. Government of the Kingdom of eSwatini Ministry of Health, 2018.
46. Consolidated Guidelines for Prevention and Treatment of HIV in Uganda Ministry of Health, Republic of Uganda, 2016.
47. National Policy Guidelines on HIV AND AIDS. Government of Gambia: National AIDS Council, 2014.
48. Malawi HIV Tetsing Services Guidelines. Government of Malawi: Ministry of Health, 2016.
49. Integrated Guidelines for Prevention, Testing, Care and Treatment of HIV and AIDS in Liberia. Republic of Liberia: Ministry of Liberia, 2015.
50. National Guidelines for Antiretroviral Therapy. Government of the Republic of Namibia Ministry of Health and Social Services, 2016.
51. National Guidelines for Comprehensive HIV Prevention, Care and Treatment: Federal Ministry of Health, Ethiopia, 2017.
52. Kenya HIV Testing Services Guidelines: National AIDS Control Programme 2015.
53. Politique, Normes et Protocoles des Services Depistage du VIH: Republique du Sénégal Ministere de la Sante Publique, 2017.
54. Somalia HIV Testing and Counseling Policy: Ministry of Health, 2017.
55. UNAIDS. United Republic of Tanzania lowers age of consent for HIV testing Geneva: United Nations Joint Programme on HIV/AIDS; 2019 [Available from:

For peer review only

https://www.unaids.org/en/resources/presscentre/featurestories/2019/november/united-republic-of-tanzania-lowers-age-of-consent-for-hiv-testing accessed 7 July 2020.
56. Directives technique de prevention du sida et de pris en charge de personnes vivant avec le VIH. Abidjan: Ministere de la sante et de l'hygiene, 2019.
57. Differentiated service delivery for HIV in Ghana: an operational manual National AIDS Control Programme, 2017.
58. HIV Testing Services National Guidelines: Ministry of Health, Republic of Zambia, 2016.
59. Zimbabwe National Guidelines on HIV Testing and Counselling, second edtion. In: Care MoHaC, ed., 2014.
60. Strategie Nationale de Demedicalisation du Depitage du VIH au Benin, 2017.
61. Normes et Directives Nationales de Conseil Depistage de L'infection a VIH au Burkina Faso. Gouvernement de Burkina Faso: Ministere de la Sante, 2008.
62. Guide du Depistage et Conseil du VIH la L'initiative du Prestataire. Gouvernement de la Republic Centrafricaine Ministere de la Sante Publique, 2010.
63. Eritrea National Guidelines for HIV Testing Services. Asmara: Ministry of Health, Deptartment of Public Health, Communicable Diseases Control Division, National AIDS Control Program, 2019.
64. Counseling et Depistage du VIH a Madagasca Rnormes et Procedures. Republic of Madagascar: Le Ministre de la Santé Publique, 2011.
65. Normes et Procedures des Services de Despistage du VIH au Mali: Haut Conseil National de Lutte Contre le SIDA, 2017.
66. Guidelines for HIV Tetsing Services in Sierra Leone National AIDS Control Programme: Ministry of Health and Sanitation, 2017.
67. Consolidated Clinical Guidelines on Use of Antiretroviral Drugs for HIV Treatment and Prevention Government of the Republic of South Sudan: Ministry of Health, 2017.
68. Directives Nationales D'utilisation des Antiretroviraux Pour la Prevention et le Traitement du VIH. Gouvernement de la Republique du Burundi: Ministere de la Sante, 2016.
69. Document de Politique,Normes et Procedures des Services de Depistage du VIH en Côte D'ivoire. Le gouvernement de Cote D'Ivoire: Ministère en charge de la Santé et de l'Hygiene Publique, 2016.
70. National HIV and AIDS Policy: Republic of Mauritius, 2010.
71. National Guidelines for the Management of HIV and AIDS. Government of the United Republic of Tanzania: Ministry of Health, Community Development, Gender, Elderly, and Children 2017.
72. Lesotho National Policy & Guidelines on HIV Testing Services: Ministry of Health Lesotho, 2017.
73. Guidelines for Antiretroviral Therapy for the Prevention and Treatment of HIV in Zimbabwe, 2016: Ministry of Health and Child Care, 2016.
74. Torres M, Gruskin S, Buse K, et al. Monitoring HIV-Related Laws and Policies: Lessons for AIDS and Global Health in Agenda 2030. *AIDS Behavior* 2017;21:S51–S61. doi: https://doi.org/10.1007/s10461-016-1621-5
75. Jamil M, Eshun-Wilson I, Charles Witzel T*ea*. Effectiveness of HIVST Distribution Models in the General Population in SSA: A Systematic Review [abstract]. International Conference on AIDS and STIs in Africa. Kigali, , 2019.
76. OraQuick® in-home HIV test summary of safety and effectiveness. Washington, DC: United States Food and Drug Administration, 2012.
77. Staveteig S, Croft T, Kampa K, et al. Reaching the 'first 90': Gaps in coverage of HIV testing among people living with HIV in 16 African countries. *PloS one* 2017;12(10):e0186316. doi: https://doi.org/10.1371/journal.pone.0186316

For peer review only

Search Strategy

The process for collecting HTS guidelines included the following methods:

1. Search of internal WHO database for HIV Testing Services (HTS) policy documents

2. Country by country search of AIDSFree HTS policy database (https://aidsfree.usaid.gov/resources/guidance-data/hts)

3. Country by country search of IAPAC/HIV Policy Watch website (http://www.hivpolicywatch.org/database.html)

4. Broad Google search for HTS policies from each WHO country using the following key words
in English, French, Portuguese, and Spanish:

- country name AND “HIV testing” AND policy
- country name AND “HIV testing” AND guideline
- country name AND PrEP AND policy
- country name AND PrEP AND guideline
- country name AND “pre-exposure prophylaxis” AND policy
- country name AND “pre-exposure prophylaxis” AND guideline

The policy repository is maintained by WHO.

For peer review only

Policy data extraction tool

Two reviewers extracted policy data using a standardised tool. The tool included the following:

1. Country name
2. WHO priority country (yes/no)
3. Sub-region (ESA, WCA)
4. HIV testing policy identified? /yes/no, for HIVST policies, document separately)
    - Type of HIV testing policy document (HTS, NSP, ART, PMTCT, other)
    - Document name, primary data source
    - Notes about document and or secondary data sources
    - HIV prevalence (≥5%, <5%)
    - Year of publication
    - Language
5. HTS policy classification
    - Law or policy existing for HTS, age of consent clearly defined, including no age limit (0/1)
    - Law or policy existing for HTS, but no age of consent specified (0/1)
    - Law or policy existing for HTS, but no mention of ‘adolescent’ or ‘minor’ (0/1)
    - No law or policy for HTS
    - Age of minor not defined
    - No age limit – all or most adolescents eligible
6. Age of consent for HIV testing (including policy page reference)
7. Exceptions
    - Shows maturity and understanding of the process and potential results, health-care provider discretion
    - At risk of contracting HIV
    - Symptomatic
    - Pregnant
    - Parent, adolescent is already a parent
    - Head of household
    - Married
    - Commercial sex worker
    - Street child
    - Emancipated minor
    - Notes concerning exceptions

8. HIVST policy identified? (yes/no)
    - Type of HIVST policy doc (HTS, NSP, ART, other)
    - HIVST Document name (primary HIVST data source)
    - Notes about HIVST document and or secondary HIVST data source
    - Year of publication
    - Language
9. HIVST policy classification
    - HIVST law or policy existing, age of consent clearly defined (including no age limit)
    - HIVST law or policy existing, but no age of consent specified for HIVST
    - HIVST law or policy existing, but no mention of ‘adolescent’ or ‘minor’
    - No law or policy for HIVST
    - No age limit – all or most adolescents eligible for HIVST
10. Age of consent for HIVST (including page reference)
11. Notes and exceptions for age of consent to HIVST
12. Age of consent as reported to NCPI (http://lawsandpolicies.unaids.org/)

For peer review only

SUPPLEMENTARY FILES

Included policies

Age of consent to HIV testing policies in 49 sub-Saharan African countries

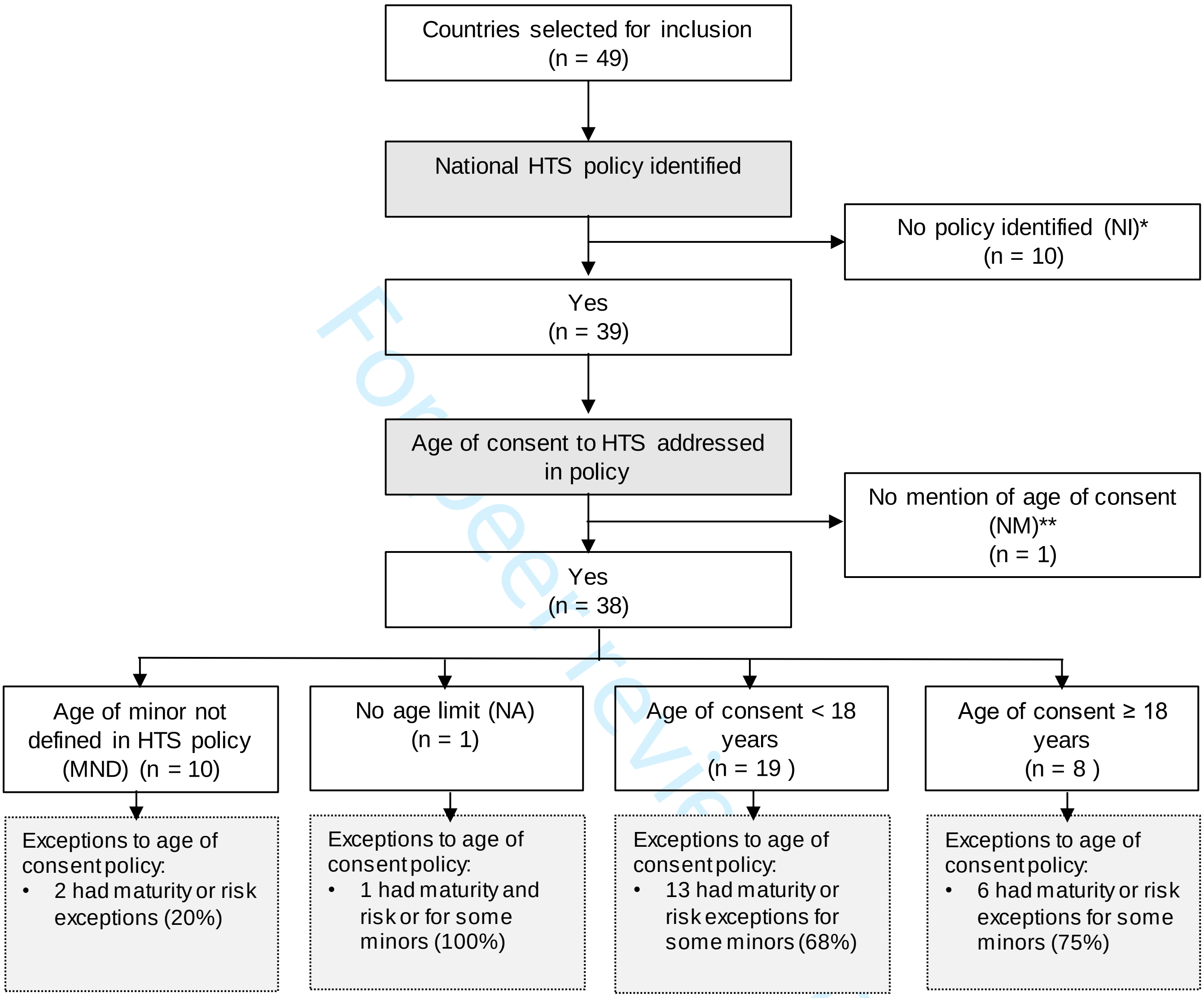

For peer review only
